# HMMR^+^ stromal cells drive human uterine development

**DOI:** 10.1038/s41421-026-00921-5

**Published:** 2026-09-08

**Authors:** Jiahui Qi, Xiaoqiang Sheng, Shiyuan Li, Peipei Jiang, Huiyan Wang, Lingxi Li, Jidong Zhou, Yifan Li, Huidan Zhang, Miaomiao Wang, Yang Zhang, Guangfeng Zhao, Guijun Yan, Bruno Péault, Yali Hu, Haixiang Sun, Lijun Ding

**Affiliations:** 1 Center for Reproductive Medicine and Obstetrics and Gynecology, Nanjing Drum Tower Hospital, Clinical College of Nanjing Medical University, Nanjing, Jiangsu China; 2 Center for Reproductive Medicine and Obstetrics and Gynecology, Nanjing Drum Tower Hospital, Affiliated Hospital of Medical School, Nanjing University, Nanjing, Jiangsu China; 3 Center for Molecular Reproductive Medicine, Nanjing University, Nanjing, Jiangsu China; 4 Department of Vascular Surgery, Cardiovascular Medical Center, Jiangsu Key Laboratory for Cardiovascular Information and Health Engineering Medicine, Nanjing Drum Tower Hospital, Affiliated Hospital of Medical School, Nanjing University, Nanjing, Jiangsu China; 5 Center for Reproductive Medicine and Obstetrics and Gynecology, Nanjing Drum Tower Hospital, Clinical College of Nanjing University of Chinese Medicine, Nanjing, Jiangsu China; 6 MRC Center for Regenerative Medicine and Center for Cardiovascular Science, University of Edinburgh, Edinburgh, Scotland UK; 7 Orthopedic Hospital Research Center and Broad Stem Cell Center, David Geffen School of Medicine, University of California, Los Angeles, CA USA; 8 State Key Laboratory of Analytic Chemistry for Life Science, Nanjing University, Nanjing, Jiangsu China; 9 Clinical Center for Stem Cell Research, Nanjing Drum Tower Hospital, Affiliated Hospital of Medical School, Nanjing University, Nanjing, Jiangsu China

**Keywords:** Mesenchymal stem cells, Stem cells

## Abstract

Early uterine development in humans is a critical determinant of reproductive health and disease susceptibility during adolescence. While single-cell analyses have extensively characterized the adult uterus, the developmental origins of this organ remain poorly understood. In this study, single-cell RNA sequencing and spatial transcriptomics of the human uterus from gestational week 12 (GW12) to GW24 and from adolescents revealed a population of hyaluronan-mediated motility receptor-expressing (HMMR^+^) stromal cells essential for uterine endometrial and myometrial development. These HMMR^+^ stromal cells were highly proliferative and differentiated into stromal- and muscle-like lineages upon in vitro induction. Following xenotransplantation into immunodeficient mice, they engrafted and formed mesenchymal-like tissue expressing both stromal and smooth muscle markers. We determined that HMMR^+^ stromal cells promote epithelial cell differentiation and development through pleiotrophin signaling. Notably, these HMMR^+^ stromal cells were significantly reduced in the thin endometrium. Collectively, our findings define the cellular ontogeny of the human uterus and identify a pivotal stromal progenitor-like population with implications for developmental disorders and endometrial pathologies.

## Introduction

The human uterus is a unique organ capable of scar-free regeneration through ~400 cycles of remodeling during a woman’s reproductive lifespan^1^. Its development is a highly orchestrated process that begins early in embryogenesis, continues throughout gestation, and ultimately achieves functional maturity at menarche^2^. Following the formation of Müllerian ducts around the sixth gestational week (GW6) and their caudal fusion around GW8, the three principal layers of the uterus — the endometrium, myometrium, and perimetrium — differentiate and organize during fetal development^3^. A comprehensive understanding of the cellular and molecular mechanisms governing this process is essential for elucidating both normal physiology and the pathogenesis of reproductive disorders^4^.

Recent advances in single-cell RNA sequencing (scRNA-seq) and spatial transcriptomics have revolutionized the study of cellular heterogeneity with unprecedented resolution^5^. While most developmental scRNA-seq studies rely on rodent models and focus on adult tissues, fetal human samples provide unparalleled insights into organogenesis^6–8^. The endometrial epithelium differentiates from the undifferentiated Müllerian epithelium at GW9, glands lined with simple columnar epithelium appear at GW14, and the glands become elongated and branched within the stroma until GW21^9–11^. In adulthood, this epithelium comprises distinct subtypes, including SOX9^+^ cells, LGR5^+^ luminal cells, SCGB2A2^+^ glandular cells, and PIFO^+^TPPP^+^ ciliated cells^12^. Concurrently, the loose mesenchyme around the Müllerian ducts begins to differentiate into stromal and muscular layers by GW8^13^. Smooth muscle becomes more prominent by GW11–12, establishing structural integrity^14^. As gestation progresses, the inner stromal layer develops into richly vascularized and hormone-responsive tissue and interacts with the epithelium, facilitating glandular formation and overall uterine cytoarchitecture^15^. The outer stromal layer gives rise to organized myometrium after GW20^14^.

Several key regulators of stromal development have been identified during postnatal uterine development and the adult menstrual cycle. Ablation of Pr-set7 activates interferon signaling, induces cellular apoptosis, and results in stromal malformations^16^. In addition, the SFRP4^+^ stromal subpopulation supports the proliferation of epithelial glands and promotes endometrial repair through the IGF1 signaling pathway in the adult uterus^17^. Given the central role of stromal cells in regulating uterine functions, it is crucial to define their developmental trajectory, identify their progenitor components, and elucidate the signals that guide epithelial and muscle differentiation.

In this study, we employed scRNA-seq and spatial transcriptomics to map the developmental trajectories of epithelial cells, stromal cells, and smooth muscle cells (SMCs) in the human fetal uterus from GW12 to GW24, validating the dynamic changes of cell-specific markers. Herein, we identified a unique proliferative population of hyaluronan-mediated motility receptor-expressing (HMMR^+^) stromal cells with multipotent stromal cell functions. Under both in vitro and in vivo inductive conditions, HMMR^+^ stromal cells exhibited the potential to differentiate into mesenchymal and myogenic lineages. These findings raise the possibility that HMMR^+^ stromal cells may contribute to endometrial and myometrial development. Notably, this cell population was markedly reduced in samples from individuals with thin endometrium, suggesting a potential link between HMMR^+^ stromal cell deficiency and the pathogenesis of endometrial dysfunction.

## Results

### Cellular atlas of the human fetal uterine corpus

**Fig. 1:**
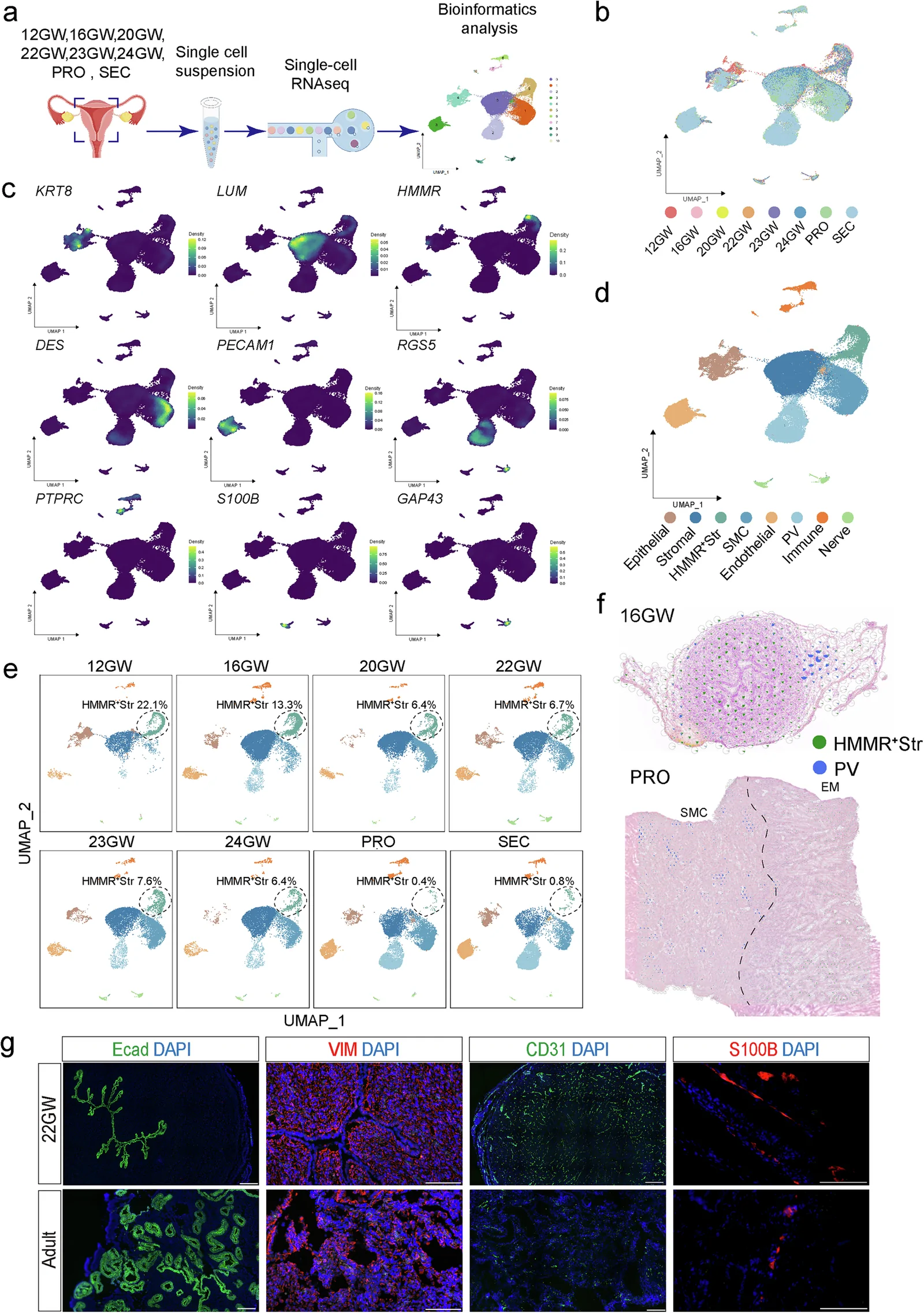
Single-cell transcriptomics of human fetal and adult uteri. **a** Sample source and scRNA-seq workflow (by Figdraw 2.0). **b** Uniform Manifold Approximation and Projection (UMAP) plot of all uterine cells colored by developmental stage. **c** Plot density depicting classical specific markers for uterine cell populations (*KRT8* for epithelial cells; *LUM* for stromal cells; *HMMR* for HMMR^+^ stromal cells; *DES* for SMCs; *PECAM1* for endothelial cells; *RGS5* for perivascular cells; *PTPRC* for immune cells; *S100B* and *GAP43* for neural cells). **d** UMAP plot of cells with the associated cell types in full-thickness uterine samples. HMMR^+^ Str, HMMR^+^ stromal cells; SMC, smooth muscle cells; PV, perivascular cells; Nerve, neural cells. **e** UMAP plot showing cells from the full uterus at different developmental stages. **f** Spatial mapping of HMMR^+^ Str and PV cells in a transverse section of the fetal uterus (GW16) and a full-thickness uterine section from the PRO using RCTD deconvolution of Visium spatial transcriptomics data. The color filling proportion indicates the predicted percentage of each cell type within individual Visium spots. **g** Immunofluorescence for E-cadherin (epithelium), vimentin (stroma), CD31 (endothelial cells), and S100B (neural cells) in GW22 and adult uteri. GW gestational weeks, PRO proliferative phase, SEC secretory phase. Scale bars, 100 μm.

To construct a cellular map of uterine development, we performed scRNA-seq on entire uterine corpus samples from fetuses (GW12, GW16, GW20, GW22, GW23, and GW24) and full-thickness uterine samples from reproductive-aged women in the proliferative and secretory phases (Fig. 1a; Supplementary Fig. S1a). We generated a transcriptomic map comprising 93,662 cells, which segregated into 11 clusters (Fig. 1b). Furthermore, these clusters were assigned identities as eight major cell types on the basis of canonical markers: (1) epithelial cells marked by *KRT8* and *E-cadherin*; (2) stromal cells marked by *LUM*, *DCN*, and *VIM*; (3) HMMR^+^ stromal cells (HMMR^+^ Str), defined by stromal cell-associated genes and high expression of proliferation markers, including *HMMR*, *TOP2A* and *Ki67*; (4) SMCs marked by *DES* and *ACTG2*; (5) endothelial cells marked by *PECAM1*, *EMCN*, and *ADGRL4*; (6) perivascular cells (PV cells) marked by *RGS5* and *RCAN2*; (7) immune cells (clusters 6, 7, and 10) specifically expressing *PTPRC*; and (8) neural cells (clusters 8 and 9) expressing glial (*S100B*) and neuronal (*GAP43*) markers (Fig. 1c, d; Supplementary Fig. S1b).

In fetal uteri (GW12–22), epithelial cells, stromal cells, and HMMR^+^ stromal cells constituted the predominant cellular components. In contrast, adult uteri were characterized by a relative enrichment of vascular-associated cell types. Specifically, endothelial and PV cells, which together form the uterine vasculature, became prominent (Fig. 1e; Supplementary Fig. S1c). Notably, the number of HMMR^+^ stromal cells markedly decreased during this maturational transition (Fig. 1e; Supplementary Fig. S1c). Spatial deconvolution was used to map the spatiotemporal distribution of these cell types across key developmental stages (GW16, GW20, and proliferative and secretory phases) (Supplementary Fig. S1d). HMMR^+^ stromal cells were broadly disseminated within the fetal uterine stroma (GW16/20), whereas in adulthood, PV cells formed prominent perivascular clusters around the endometrial and myometrial vessels (Fig. 1f).

The results of histological and immunofluorescence analyses corroborated these transcriptomic findings. At GW22, hematoxylin and eosin (H&E) and E-cadherin staining revealed that the uterine epithelium consisted of a simple columnar epithelium with invaginating glands, surrounded by dense stromal cells. The adult epithelium featured abundant glandular epithelium dispersed within the stromal cells (Fig. 1g; Supplementary Fig. S1e). Vimentin staining confirmed a denser stromal architecture at GW22 compared with the looser arrangement in adult uteri (Fig. 1g; Supplementary Fig. S1f). CD31 staining revealed a dispersed vascular network in the fetal endometrium (GW22), in contrast to the more concentrated, mature vasculature in adult tissue (Fig. 1g). The neural cell marker S100B was localized near the uterine basement membrane in the fetal stages (GW16/20), but was sparsely expressed in the adult endometrium (Fig. 1g; Supplementary Fig. S1g). The proliferative marker Ki67 was widely expressed in the uterine endometrium (GW22) but was significantly reduced in adulthood (Supplementary Fig. S1h).

To further validate our results, we analyzed a public fetal uterine scRNA-seq dataset^5^ across seven gestational stages (GW10, GW12, GW13, GW15, GW18, GW20, and GW21) (Supplementary Fig. S2a). Unsupervised clustering of these data resolved 15 distinct clusters (Supplementary Fig. S2b). Each of the major cell types identified in our study — including epithelial cells, stromal cells, HMMR^+^ stromal cells, SMCs, endothelial cells, and PV cells — was reliably recapitulated in this public dataset (Supplementary Fig. S2c, d). Quantification of cell type proportions across the selected stages revealed a progressive decrease in the number of HMMR^+^ stromal cells with increasing gestational age (from GW10 to GW21) (Supplementary Fig. S2e), mirroring the developmental decrease observed in our primary dataset (Fig. 1e). Spatial transcriptomics of fetal uterus at GW18 from the same public dataset further revealed that HMMR^+^ stromal cells were predominantly localized in the stromal compartment (Supplementary Fig. S2f). Collectively, these independent analyses support the robustness of our cell type annotations and confirm that the HMMR^+^ stromal cell population is reproducibly detectable across datasets.

Overall, these dynamic changes reflect the transition of the human uterus from the formation of its basic structure in the fetus to functional specialization and maturation in adults.

### HMMR^+^ stromal cells in the fetal uterus

**Fig. 2:**
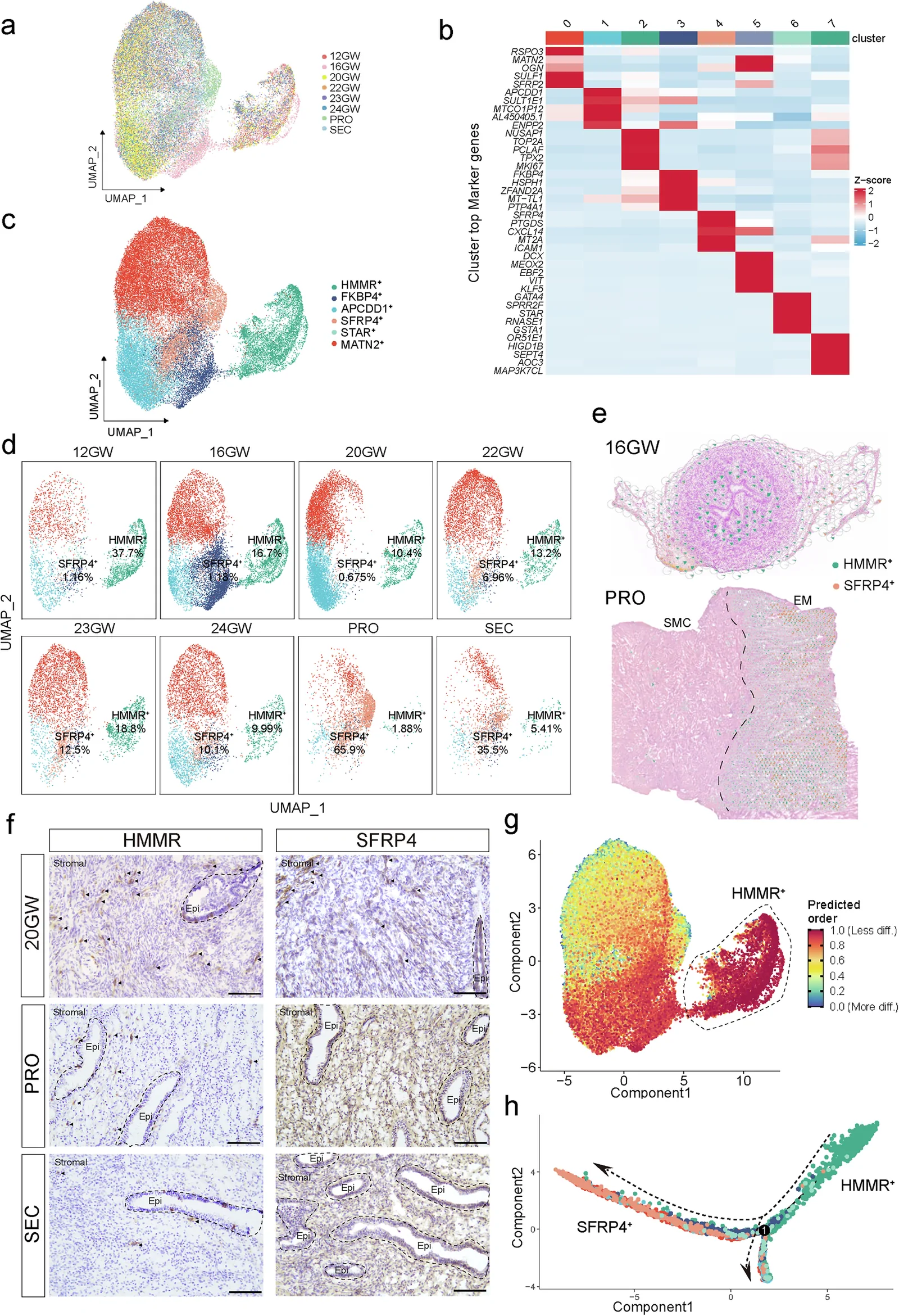
Proportions and trajectories of uterine stromal cells of human fetal and adult uteri. **a** UMAP plot of all uterine stromal cells colored by developmental stage. **b** Heatmap showing the top 5 specifically expressed genes for stromal cell subpopulations. **c** UMAP plot of the six main stromal cell subpopulations. **d** UMAP plot showing stromal cell subpopulations at different developmental stages. **e** Spatial mapping of the HMMR^+^ stromal subpopulation and SFRP4^+^ stromal subpopulation in a transverse section of the fetal uterus (GW16) and a full-thickness uterine section from the PRO using RCTD deconvolution of Visium spatial transcriptomic data. The color filling proportion indicates the predicted percentage of each cell type within individual Visium spots. **f** Immunohistochemistry staining of human uterine sections reveals the expression of HMMR and SFRP4 at GW20 and during the adult proliferative and secretory phases. Scale bars, 100 μm. Arrows: positive cells detected by immunohistochemical staining. **g** UMAP plot showing CytoTRACE differentiation state scores in each stromal cell subpopulation. **h** Monocle2 pseudotime trajectory analysis of stromal cell subpopulations. GW gestational weeks, PRO proliferative phase, SEC secretory phase.

While single-cell transcriptomics has characterized the adult uterus, the developmental trajectory of human uterine mesenchymal compartments from the fetus to the adult remains poorly defined^12,17^. Herein, human endometrial stromal cells (including HMMR^+^ stromal cells) from eight fetal and adult samples were classified into eight subpopulations (Fig. 2a, b). Based on distinct marker gene expression and gene ontology analyses, we identified six primary stromal subtypes: HMMR^+^, FKBP4^+^, APCDD1^+^, SFRP4^+^, STAR^+^, and MATN2^+^ (Fig. 2b, c; Supplementary Fig. S3a). HMMR^+^ stromal cells (clusters 2 and 7) exhibited a high expression of key genes involved in cell proliferation and division, including *NUSAP1*, *TOP2A*, *PCLAF*, *TPX2*, and *Ki67* (Fig. 2b; Supplementary Fig. S3b). FKBP4^+^ stromal cells presented elevated expression of *FKBP4*, *HSPH1*, *ZFAND2A*, *MT-TL1*, and *PTP4A1*, genes critical for protein homeostasis, stress response, energy metabolism, and signal transduction^18^. APCDD1^+^ stromal cells exhibited increased expression of genes involved in regulating cell proliferation and hormone metabolism^19^ (*APCDD1*, *SULT1E1*, and *ENPP2*) and were enriched in protein synthesis processes (Fig. 2b; Supplementary Fig. S3b). SFRP4^+^ stromal cells, a previously identified regenerative adult subpopulation, were also present^17^. STAR^+^ stromal cells (expressing *SPRR2F*, *STAR*, and *GATA4*) were predominantly found at GW12 (Fig. 2b–d; Supplementary Fig. S3c), as confirmed by STAR staining (Supplementary Fig. S3d). MATN2^+^ stromal cells (clusters 0 and 5) specifically expressed *MATN2*, *OGN*, and *SULF1* (Fig. 2b, c). These cells composed a fundamental stromal subpopulation involved in the formation and regulation of the extracellular matrix (Fig. 2c, d; Supplementary Fig. S3b). Additionally, the spatial distribution of these stromal subpopulations was also deconvoluted from the spatial transcriptomics across the GW16, GW20, and proliferative and secretory phases (Supplementary Fig. S3e).

Our results revealed that HMMR^+^ stromal cells were highly enriched during the fetal stage but decreased markedly in adulthood (Fig. 2d; Supplementary Fig. S3c). In contrast, SFRP4^+^ stromal cells steadily increased with development and ultimately became the dominant adult stromal subtype (Fig. 2d; Supplementary Fig. S3c). Spatial transcriptomics confirmed this reciprocal distribution of HMMR^+^ and SFRP4^+^ stromal cells at GW16 and during the adult proliferative phase (Fig. 2e). Immunohistochemical analysis revealed that HMMR expression was enhanced in fetal (GW20) stroma relative to that in the adult proliferative and secretory phases. In contrast, SFRP4 was widely expressed in adult but not fetal stromal cells (Fig. 2f). Cell cycle analysis indicated that HMMR^+^ stromal cells were predominantly in the G2/M and S phases, highlighting their active proliferative potential (Supplementary Fig. S4a). Immunofluorescence analysis also revealed the co-localization of HMMR with Ki67 in adult tissue (Supplementary Fig. S4b).

To define stromal cell fate, trajectory inference (Monocle2) and stemness prediction (CytoTRACE) analyses revealed that HMMR^+^ stromal cells possessed greater potential to differentiate into multiple cell types (Fig. 2g) and suggested a differentiation pathway from HMMR^+^ to SFRP4^+^ stromal cells (Fig. 2h; Supplementary Fig. S4c). Spatial transcriptomic analysis revealed that *HMMR* expression was predominantly localized within the stroma compartment (Supplementary Fig. S4d). Immunohistochemical staining revealed that HMMR^+^ stromal cells were broadly distributed in the fetal stroma but became restricted near the luminal epithelium and were sparse in the basal layer in adulthood (Supplementary Fig. S4e). Notably, HMMR expression was also detected in a subset of adult epithelial cells (Supplementary Fig. S4e).

In summary, these data suggest that HMMR^+^ stromal cells represent a fetal-enriched, proliferative progenitor-like population within the human uterine mesenchyme.

### Mesenchymal differentiation of HMMR^+^ stromal cells

**Fig. 3:**
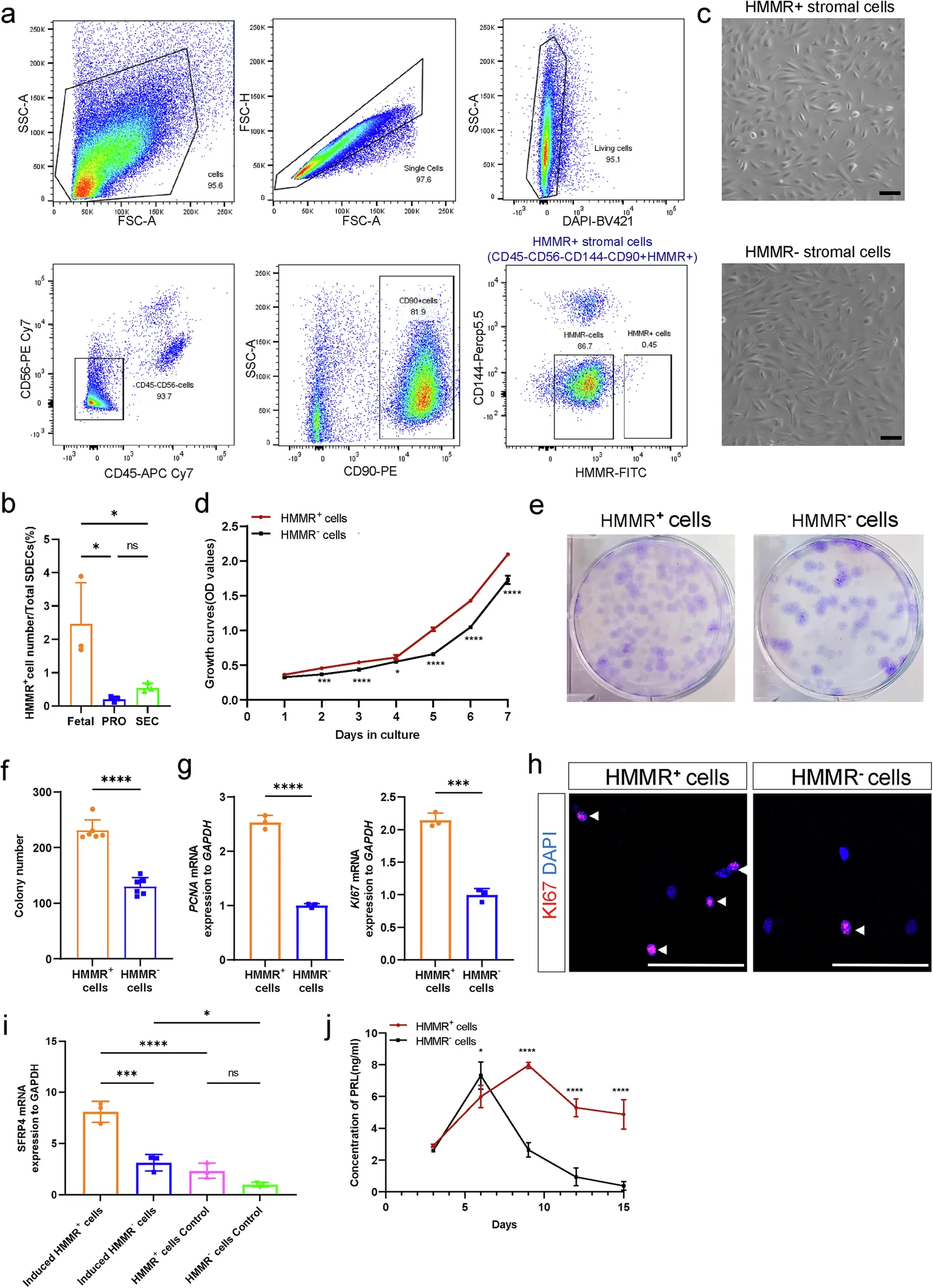
Characteristics of endometrial HMMR^+^ stromal cells. **a** Sorting strategy for stromal cell subpopulations. HMMR^+^ stromal cells (CD45^–^CD56^–^CD144^–^CD90^+^HMMR^+^) and HMMR^–^ stromal cells (CD45^–^CD56^–^CD144^–^CD90^+^HMMR^–^) were isolated by flow cytometry. **b** Quantification of HMMR^+^ stromal cells. The proportion of HMMR^+^ stromal cells among the total SDECs was significantly greater in the fetal stage than in the adult stage (PRO and SEC). Fetal: *n* = 3; PRO: *n* = 3; SEC: *n* = 3. **c** Morphologies of the in vitro-cultured HMMR^+^ stromal cells and HMMR^–^ stromal cells, exhibiting spindle-like characteristics. Scale bars, 100 μm. **d** CCK-8 analysis of proliferation in HMMR^+^ and HMMR^–^ stromal cells (*n* = 3 per group). **e**, **f** CFU numbers in HMMR^+^ and HMMR^–^ stromal cells as determined by a colony formation assay (*n* = 6 per group). **g** Gene expression levels of proliferation-related genes (*PCNA* and *Ki67*) normalized to those of *GAPDH* in HMMR^+^ and HMMR^–^ stromal cells (*n* = 3 per group). **h** Immunofluorescence staining of Ki67 in HMMR^+^ and HMMR^–^ stromal cells. Scale bars, 100 μm. Arrows: positive cells detected by immunofluorescence staining. **i** Gene expression levels of *SFRP4* before and after induction normalized to those of *GAPDH* in HMMR^+^ and HMMR^–^ stromal cells (*n* = 3 per group). **j** PRL concentration in the culture supernatant after the induction culture of HMMR^+^ and HMMR^–^ stromal cells (*n* = 3 per group). Data are expressed as mean ± standard deviation (SD). *P < 0.05, **P < 0.01, ***P < 0.001, ****P < 0.0001, ns not significant. Data in **b**, **j** were analyzed using one-way ANOVA with Tukey’s multiple comparisons test. Data in **d**, **f**, **g**, **i** were analyzed using an unpaired *t*-test.

Flow cytometric analysis revealed that CD45^–^CD56^–^CD144^–^CD90^+^HMMR^+^ stromal cells constituted 2.46% ± 1.24% of the cells in the fetal uterine endometrium, a proportion significantly greater than that in the adult proliferative (0.2% ± 0.09%, *P* < 0.05) or secretory (0.54% ± 0.14%, *P* < 0.05) phases (Fig. 3a, b). To investigate the role of HMMR^+^ stromal cells in human uterine development, we isolated CD45^–^CD56^–^CD144^–^CD90^+^ stromal cells that were HMMR^+^ or HMMR^–^ from adult endometrial samples obtained during the proliferative and secretory phases. In vitro, HMMR^+^ and HMMR^–^ stromal cells displayed a spindle-shaped morphology (Fig. 3c; Supplementary Fig. S5a, b). Immunofluorescence analysis confirmed that these cultured cells expressed the stromal cell marker vimentin and did not express the epithelial cell marker E-cadherin (Supplementary Fig. S5c, d). Both cell types also exhibited a mesenchymal stem cell (MSC) phenotype expressing CD13, CD29, CD44, CD73, CD90, and CD105 but not CD34, CD45, CD144, or HLA-DR (Supplementary Fig. S5e, f). These results indicate that both populations possess multipotent stromal cell properties.

However, HMMR^+^ stromal cells exhibited greater proliferative capacity. A CCK-8 assay revealed higher proliferation rates for HMMR^+^ stromal cells (4.77 ± 0.05) than for HMMR^–^ stromal cells (4.25 ± 0.11) by Day 7 (*P* < 0.0001; Fig. 3d). The HMMR^+^ stromal cells also formed significantly more colony-forming units (CFUs) (230.70 ± 19.18) compared with the HMMR^–^ stromal cells (130.20 ± 15.92, *P* < 0.0001; Fig. 3e, f). Similarly, compared with the HMMR^–^ stromal cells, cultured HMMR^+^ stromal cells exhibited 1.1-fold and 1.5-fold increases in *Ki67* and *PCNA* expression, respectively (Fig. 3g), which was further confirmed by Ki67 immunofluorescence (Fig. 3h).

We next induced the differentiation of both cell types toward a mature SFRP4^+^ stromal lineage using a defined differentiation medium^20^. After 21 days of induction, SFRP4 expression was elevated in both cell types, with levels in differentiated HMMR^+^ stromal cells being 1.6-fold higher than those in differentiated HMMR^–^ stromal cells (Fig. 3i). Moreover, after 15 days of induction, HMMR^+^ stromal cells secreted significantly more prolactin (PRL) — a marker of decidualization — than HMMR^–^ stromal cells (4.88 ± 0.92 vs 0.37 ± 0.29, *P* < 0.0001; Fig. 3j).

**Fig. 4:**
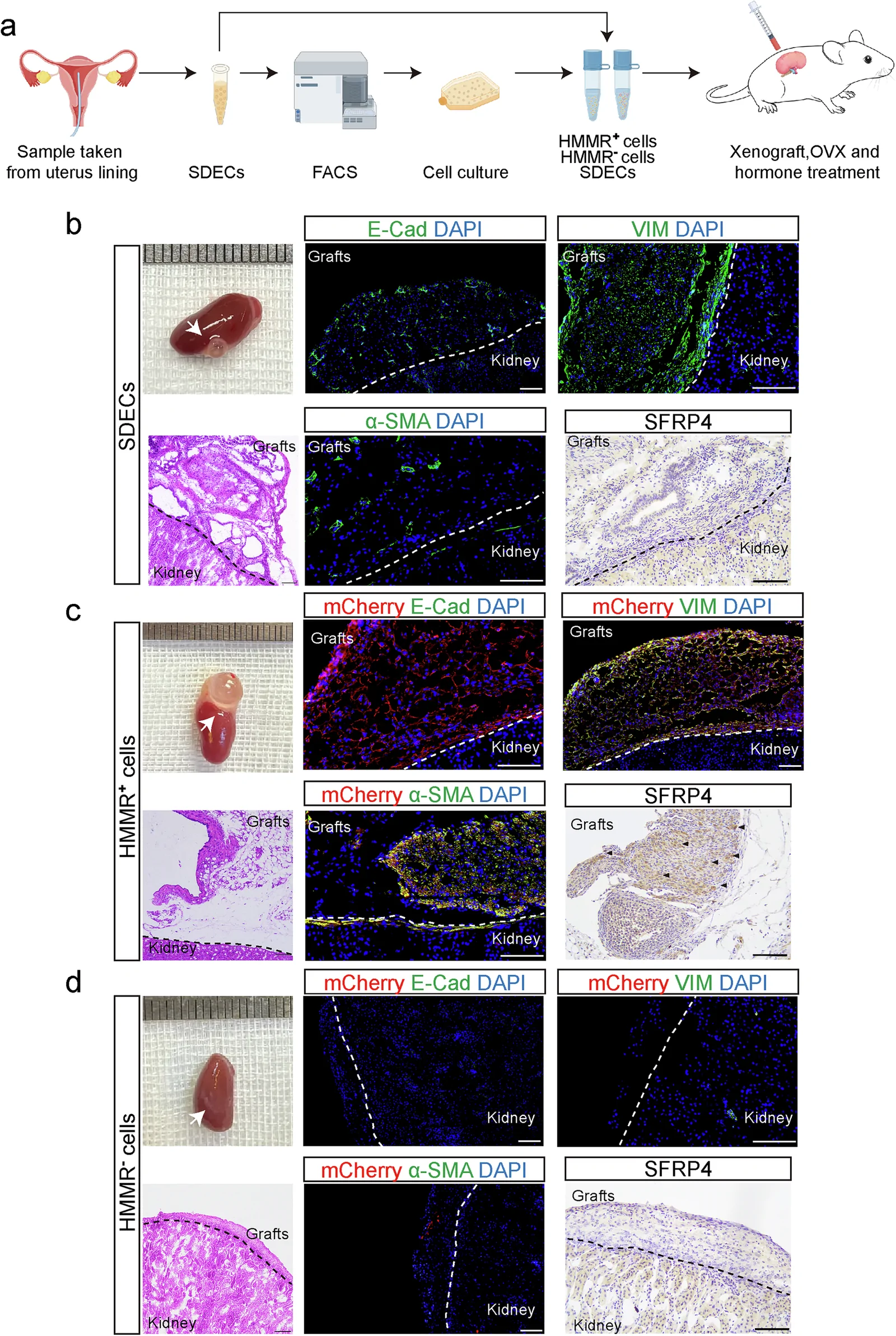
Xenotransplantation of HMMR^+^ stromal cells into the kidney capsules of immunodeficient mice. **a** Detailed workflow for xenograft transplantation (by Figdraw 2.0). **b** Macroscopic images, H&E staining, immunofluorescence staining for E-cadherin (epithelium), vimentin (mesenchyme), and α-SMA (smooth muscle), and immunohistochemical staining for SFRP4 in the kidneys of NCG mice in the SDEC transplantation group. Scale bars, 100 μm. **c** Macroscopic images, H&E staining, immunofluorescence staining for E-cadherin (epithelium), vimentin (mesenchyme), α-SMA (smooth muscle), and mCherry-positive stromal cells (red fluorescence), and immunohistochemical staining for SFRP4 in the kidneys of NCG mice in the HMMR^+^ stromal cell transplantation group. Scale bars, 100 μm. **d** Macroscopic images, H&E staining, immunofluorescence staining for E-cadherin (epithelium), vimentin (mesenchyme), α-SMA (smooth muscle), and mCherry-positive stromal cells (red fluorescence), and immunohistochemical staining for SFRP4 in the kidneys of NCG mice in the HMMR^–^ stromal cell transplantation group. Scale bars, 100 μm.

To assess differentiation potential in vivo, we performed xenotransplants under the kidney capsules of immunodeficient NCG mice using: (1) human single dispersed endometrial cells (SDECs), (2) mCherry-labeled cultured HMMR^+^ stromal cells, or (3) mCherry-labeled cultured HMMR^–^ stromal cells (Fig. 4a). After eight weeks of transplantation, the SDEC group recapitulated endometrial tissue structures, including E-cadherin^+^ glandular epithelium, vimentin^+^ mesenchyme, and α-SMA^+^ smooth muscle (Fig. 4b). Cystic masses derived from HMMR^+^ stromal cells were notably larger than those from HMMR^–^ stromal cells. Histological analysis revealed that tissue with human uterine mesenchymal and smooth muscle morphology, containing vimentin^+^ stromal cells, α-SMA^+^ SMCs, and mCherry-labeled cells, was only generated in the HMMR^+^ transplant group (Fig. 4c, d). Furthermore, robust SFRP4 expression was detected within the stromal compartment of the HMMR^+^-derived tissue (Fig. 4c), indicating that these cells retain the potential to differentiate into SFRP4^+^ stromal cells in vivo.

### Differentiation of HMMR^+^ stromal cells into SMCs

**Fig. 5:**
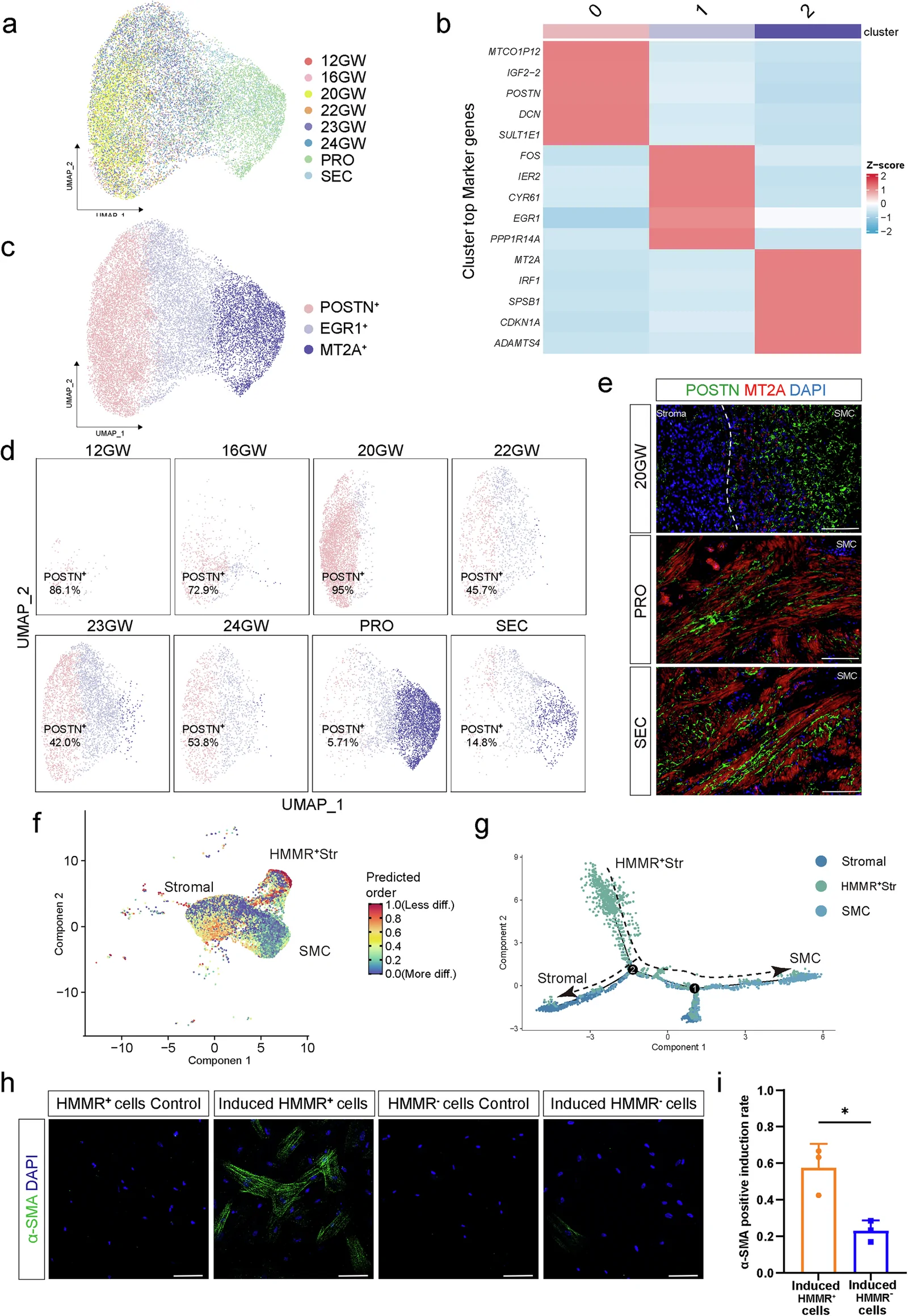
Differentiation of HMMR^+^ stromal cells into SMCs. **a** UMAP plot of all uterine SMCs colored by developmental stage. **b** Heatmap showing the top 5 specifically expressed genes in each SMC subpopulation. **c** UMAP plot of the three main SMC subpopulations. **d** UMAP plot showing SMC subpopulations at different developmental stages. **e** Immunofluorescence staining of human uterine sections reveals the expression of POSTN and MT2A at GW20 and in the adult PRO and SEC phases. Scale bars, 100 μm. **f** UMAP plot showing CytoTRACE differentiation state scores in HMMR^+^ Str, stromal, and SMCs. **g** Monocle2 pseudotime trajectory of HMMR^+^ Str, stromal, and SMCs. **h** Immunofluorescence staining of α-SMA in both HMMR^+^ and HMMR^–^ stromal cells before (control, uninduced) and after induction. Scale bars, 100 μm. **i** Induction rate of α-SMA^+^ cells. Compared with HMMR^–^ stromal cells, HMMR^+^ stromal cells exhibited a significantly greater induction rate of α-SMA^+^ cells. Data are expressed as mean ± SD. *P < 0.05, **P < 0.01, ***P < 0.001, ****P < 0.0001, ns not significant, unpaired *t*-test.

Human myometrial SMCs differentiate from the uterine mesenchyme at GW12, with a well-defined primitive myometrium by GW22^21^. As a vital element of the uterine wall, mature uterine smooth muscles exhibit contractile function. Single-cell transcriptomics of fetal and adult uteri, dimensionality reduction, and clustering of all SMCs revealed three distinct subpopulations (Fig. 5a). Based on the expression of specific marker genes and gene ontologies, these cells were identified as POSTN^+^ SMCs, EGR1^+^ SMCs, and MT2A^+^ SMCs (Fig. 5b, c; Supplementary Fig. S6a). The relative abundance of these SMC subpopulations revealed developmental stage-dependent changes. In the fetal stages, POSTN^+^ and EGR1^+^ SMCs predominated and were associated with tissue remodeling and organogenesis. In contrast, adult uteri were enriched in MT2A^+^ SMCs, which are involved in maintaining smooth muscle function through the regulation of metal ion homeostasis and protecting against oxidative stress (Fig. 5d; Supplementary Fig. S6b, c).

The dynamic changes in SMC subpopulation proportions during development aligned with spatial distributions, as revealed by deconvolution analysis of fetal (GW16) and adult (secretory phase) uteri (Supplementary Fig. S6d). Immunofluorescence staining at GW20 revealed that SMCs had not yet formed mature muscle bundles and primarily expressed POSTN along with scattered EGR1 expression (Fig. 5e; Supplementary Fig. S6e). In the adult stage, mature smooth muscle bundles predominantly expressed MT2A and EGR1 (Fig. 5e; Supplementary Fig. S6e). Taken together, these findings suggest that POSTN^+^ SMCs may represent an immature or progenitor-like state, whereas MT2A^+^ SMCs likely correspond to mature, functionally specialized SMCs.

To further investigate the cellular origin of SMCs, we conducted an integrated single-cell transcriptomic analysis of uterine stromal cells and SMCs to elucidate the developmental trajectories of these cellular subpopulations using CytoTRACE and Monocle2. The results revealed that cells with higher potential (higher CytoTRACE scores and Monocle2 trajectory root) were predominantly enriched in HMMR^+^ stromal cells (Fig. 5f) and that these cells differentiated toward stromal cells and SMCs (Fig. 5g). Notably, HMMR^+^ stromal cells were located at the origin of this differentiation trajectory (Supplementary Fig. S6f). To compare the capacity for muscular differentiation, we cultured HMMR^+^ and HMMR^–^ stromal cells in a smooth muscle induction medium^22^. Over time, the cellular morphology gradually transitioned from a spindle-shaped morphology to an elongated fusiform shape, a hallmark characteristic of SMCs (Supplementary Fig. S7a, b). Quantitative real-time PCR (qPCR) analysis revealed that following induction, HMMR^+^ stromal cells exhibited a 33.4-fold, 9-fold, and 2.3-fold increase in the expression of *α-SMA*, *calponin-1* (*CNN1*), and *SM22α*, respectively (Supplementary Fig. S7c). In contrast, HMMR^–^ stromal cells exhibited relatively low expression of *α-SMA*, *CNN1*, and *SM22α* after induction (Supplementary Fig. S7d). Western blot analysis also confirmed the increase in α-SMA expression in HMMR^+^ stromal cells after induction (Supplementary Fig. S7e). Immunofluorescence staining also revealed that the α-SMA fibers in the cytoskeleton of induced HMMR^+^ stromal cells were rearranged, forming parallel bundle structures that exhibited the typical morphology of SMCs (Fig. 5h). HMMR^+^ stromal cells presented significantly higher induction rates of α-SMA- and SM22α-positive cells (57.50% ± 13.10% and 51.8% ± 17.70%, respectively) than HMMR^–^ stromal cells (22.93% ± 5.90%, *P* < 0.05 and 8.10% ± 2.80%, *P* < 0.05, respectively) (Fig. 5i; Supplementary Fig. S7f, g). In general, these findings suggest that HMMR^+^ stromal cells possess the potential to differentiate into myometrial SMCs under in vitro induction.

### HMMR^+^ stromal cells support epithelial growth

**Fig. 6:**
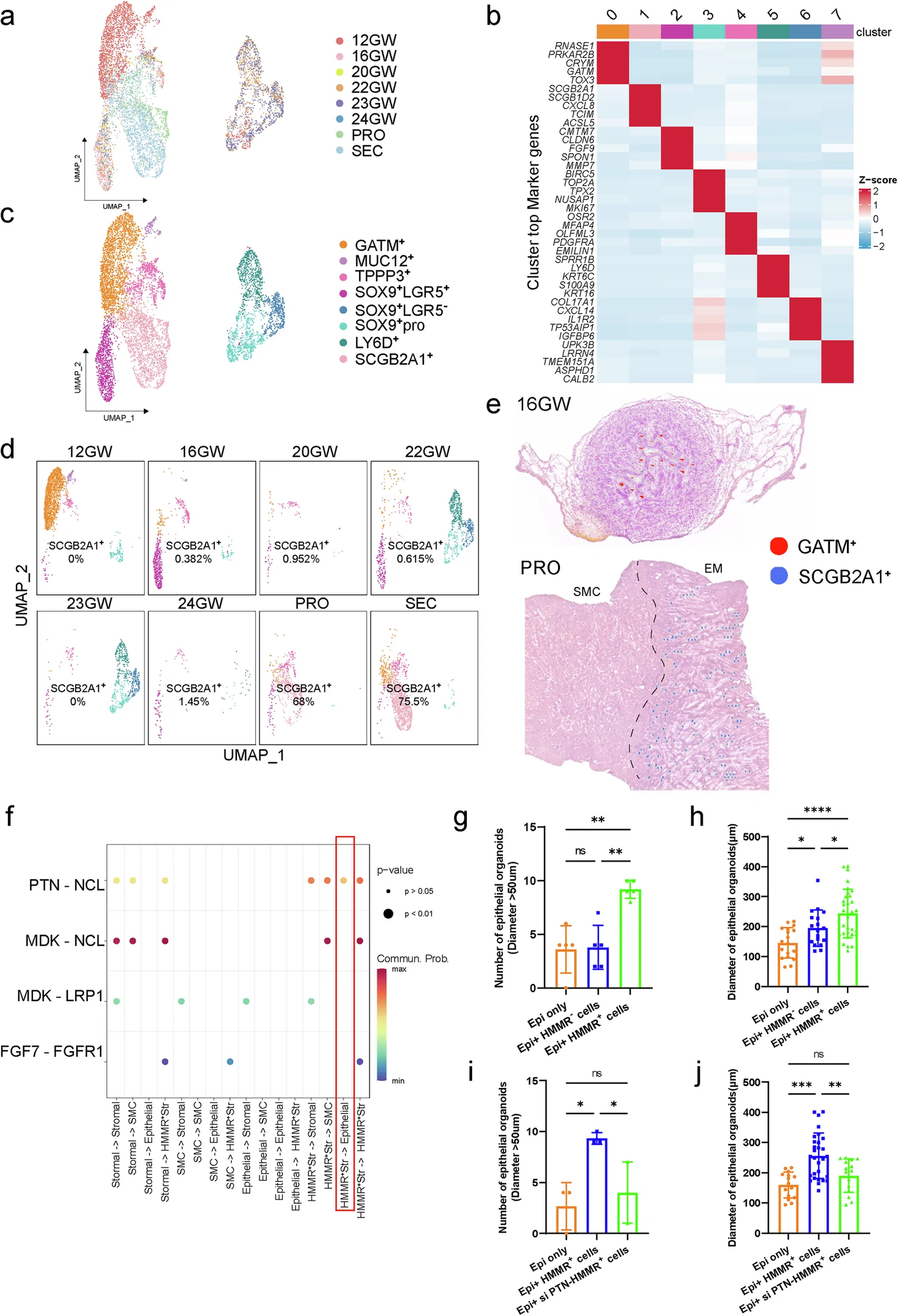
Epithelial gland growth supported by HMMR^+^ stromal cells. **a** UMAP plot of all uterine epithelial cells colored by developmental stage. **b** Heatmap showing the top 5 specifically expressed genes in each epithelial cell subpopulation. **c** UMAP plot of the eight epithelial cell subpopulations. **d** UMAP plot showing epithelial cell subpopulations at different developmental stages. **e** Spatial mapping of GATM^+^ and SCGB2A1^+^ epithelial subpopulations in a transverse section of the fetal uterus (GW16) and a full-thickness uterine section from the proliferative phase (PRO) was performed using RCTD deconvolution of Visium spatial transcriptomics data. The color filling ratio represents the predicted proportion of each cell type within individual Visium spots. **f** Ligands that are secreted and received by the stromal, SMC, epithelial, and HMMR^+^ Str cell populations in the uterus at GW20. The HMMR^+^ Str cells emit PTN signals to act on the epithelial cells. **g** Quantification of the endometrial epithelial organoids cultured with HMMR^+^ or HMMR^–^ stromal cells on Day 14 (*n* = 5 per group). **h** Diameter of the endometrial epithelial organoids cultured with HMMR^+^ or HMMR^–^ stromal cells on Day 14. **i** Quantification of the endometrial epithelial organoids cultured with control or PTN knockdown (si-PTN) HMMR^+^ stromal cells on Day 14 (*n* = 3 per group). **j** Diameter of the endometrial epithelial organoids cultured with control or PTN knockdown (si-PTN) HMMR^+^ stromal cells on Day 14. Data are expressed as mean ± SD. *P < 0.05, **P < 0.01, ***P < 0.001, ****P < 0.0001, ns not significant, one-way ANOVA with Tukey’s multiple comparisons test.

The elongation and branching of uterine epithelial glands are dependent on interactions between the epithelium and the stroma^11^. Through dimensionality reduction and clustering analysis of epithelial cell populations at different developmental stages, we identified eight distinct epithelial subpopulations on the basis of the following known markers and specific genes (Fig. 6a): (1) GATM^+^ epithelium, enriched in early development and expressing *PRKAR2B*, *CRYM*, *GATM*, and *TOX3* genes that regulate cell growth and metabolism^23^; (2) MUC12^+^ epithelium (pre-ciliated epithelium); (3) TPPP3^+^ epithelium (ciliated epithelium); (4) SOX9^+^ epithelium encompassing SOX9^+^LGR5^+^ luminal, proliferating SOX9^+^TOP2A^+^, and non-proliferating SOX9^+^LGR5^–^ epithelial subpopulations; (5) LY6D^+^ epithelium expressing *LY6D*, *S100A9*, *KRT6C*, *KRT16*, and *SPRR1B*, which are involved in epithelial stress and inflammatory responses; and (6) SCGB2A1^+^ epithelium (glandular epithelium) expressing the typical glandular markers *SCGB2A1* and *SCGB1D2* (Fig. 6b, c; Supplementary Fig. S8a)^12^.

Upon further investigation of the dynamic changes in epithelial cell subpopulations throughout uterine development, we found that the epithelium was predominantly composed of GATM^+^ epithelial cells at GW12 (Fig. 6d; Supplementary Fig. S8b). The glandular epithelium emerged during the mid-gestation period (GW16–GW20), as primitive epithelial cells differentiated into luminal and glandular cell types. The LY6D^+^ epithelial subpopulation transiently appeared between GW20 and GW24, characterized by distinct gene expression profiles indicative of epithelial development and differentiation, and was associated with epithelial differentiation and stress responses (Fig. 6d; Supplementary Fig. S8b, c). SCGB2A1^+^ epithelial maturation predominantly occurred postnatally, particularly during puberty, when the SCGB2A1^+^ epithelium became more complex and functional (Fig. 6d; Supplementary Fig. S8b). Consistent with these findings, spatial transcriptomic deconvolution revealed that GATM^+^ epithelial cells were enriched at GW16; in contrast, the adult proliferative endometrium contained abundant SCGB2A1^+^ epithelial cells (Fig. 6e). This epithelial development and maturation are essential for maintaining endometrial structure and function^21^.

To determine the fate of epithelial cells throughout uterine development, we used CytoTRACE and Monocle2 to analyze the developmental trajectories. The results revealed that GATM^+^ epithelial cells and SOX9^+^LGR5^+^ epithelial cells exhibited high differentiation potential (Supplementary Fig. S8d). Trajectory analysis further elucidated the developmental pathways of GATM^+^ and SOX9^+^LGR5^+^ epithelial cells toward the SCGB2A1^+^ epithelium (Supplementary Fig. S8e). Importantly, SOX9^+^LGR5^+^ epithelial cells have been previously identified as a potential stem cell population in the adult endometrium, whereas GATM^+^ epithelial cells represent a newly discovered immature epithelial subpopulation^24,25^. While only E-cadherin^+^ epithelial cells were detected at GW16, markers of both luminal (TROP2) and glandular (MUC1) epithelial cells became evident by GW20–22 (Supplementary Fig. S8f). Although the luminal epithelium began to navigate downward, it remained immature during mid-gestation (Supplementary Fig. S8f), which indicates that full maturation of the luminal and glandular epithelium may not be attained until adulthood.

Interactions between the uterine mesenchyme and epithelium determine luminal and glandular epithelial cell development and differentiation^11,26^. During epithelial cell development (i.e., in the fetal stage), the HMMR^+^ stromal subpopulation continuously provided high levels of pleiotrophin (PTN) to act on epithelial cells, as determined by cell–cell communication analysis (Fig. 6f; Supplementary Fig. S9a). To further explore the role of HMMR^+^ stromal cells in supporting epithelial growth and differentiation, we co-cultured HMMR^+^ and HMMR^–^ stromal cells with endometrial epithelial organoids and revealed that HMMR^+^ stromal cells significantly enhanced the proliferation of endometrial epithelial organoids after 14 days of co-culture (Supplementary Fig. S9b). Quantitative analysis revealed that the number and diameter of the organoids significantly increased in the HMMR^+^ stromal cell co-culture group (9.20 ± 0.80 and 243.90 ± 80.80 µm, respectively) compared with those of the organoids in the co-culture group with HMMR^–^ stromal cells (3.80 ± 2.00, *P* < 0.01 and 194.80 ± 60.30 µm, *P* < 0.05, respectively) or without stromal cells (3.60 ± 2.10, *P* < 0.01 and 145.20 ± 50.80 µm, *P* < 0.0001, respectively) (Fig. 6g, h). Next, we investigated whether HMMR^+^ stromal cells promoted endometrial epithelial organoid formation via PTN signaling. PTN expression was knocked down in HMMR^+^ stromal cells using small interfering RNAs (siRNAs; specifically si-PTN-3) (Supplementary Figs. S9c–e). After transfection with si-PTN-3, these cells were co-cultured with endometrial epithelial organoids (Supplementary Fig. S9f). PTN knockdown significantly impaired epithelial organoid formation, markedly reducing both the number (4.00 ± 3.00 vs 9.33 ± 0.58, *P* < 0.05) (Fig. 6i) and diameter (190.10 ± 55.16 µm vs 255.80 ± 75.13 µm, *P* < 0.01) (Fig. 6j) of the organoids compared with those in co-cultures with control HMMR^+^ stromal cells. Thus, these results revealed PTN as a key signaling molecule secreted by HMMR^+^ stromal cells that promotes endometrial epithelial organoid proliferation in vitro.

Furthermore, we performed scRNA-seq of endometrial epithelial organoids that were co-cultured with or without HMMR^+^ stromal cells. Compared with only epithelial organoids, epithelial-HMMR^+^ stromal cell co-cultured organoids contained stromal cell populations (Supplementary Fig. S9g, h). Epithelial subpopulations in the organoids were classified on the basis of marker gene expression and segregated into secretory luminal epithelium, proliferative epithelium, secretory glandular epithelium, ciliated epithelium, and immune-related epithelium (Supplementary Fig. S9i–k). Comparative analysis revealed significant alterations in epithelial composition following epithelial-HMMR^+^ stromal cell co-culture. Specifically, the proportions of secretory luminal epithelial cells and ciliated epithelial cells were increased in epithelial-HMMR^+^ stromal cell co-cultured organoids (Supplementary Fig. S9l), indicating that HMMR^+^ stromal cells not only promote organoid growth but also drive epithelial differentiation and functional maturation. These findings highlight the pivotal role of HMMR^+^ stromal cells in shaping epithelial heterogeneity and facilitating lineage specification.

### HMMR^+^ stromal cells are decreased in thin endometrium

**Fig. 7:**
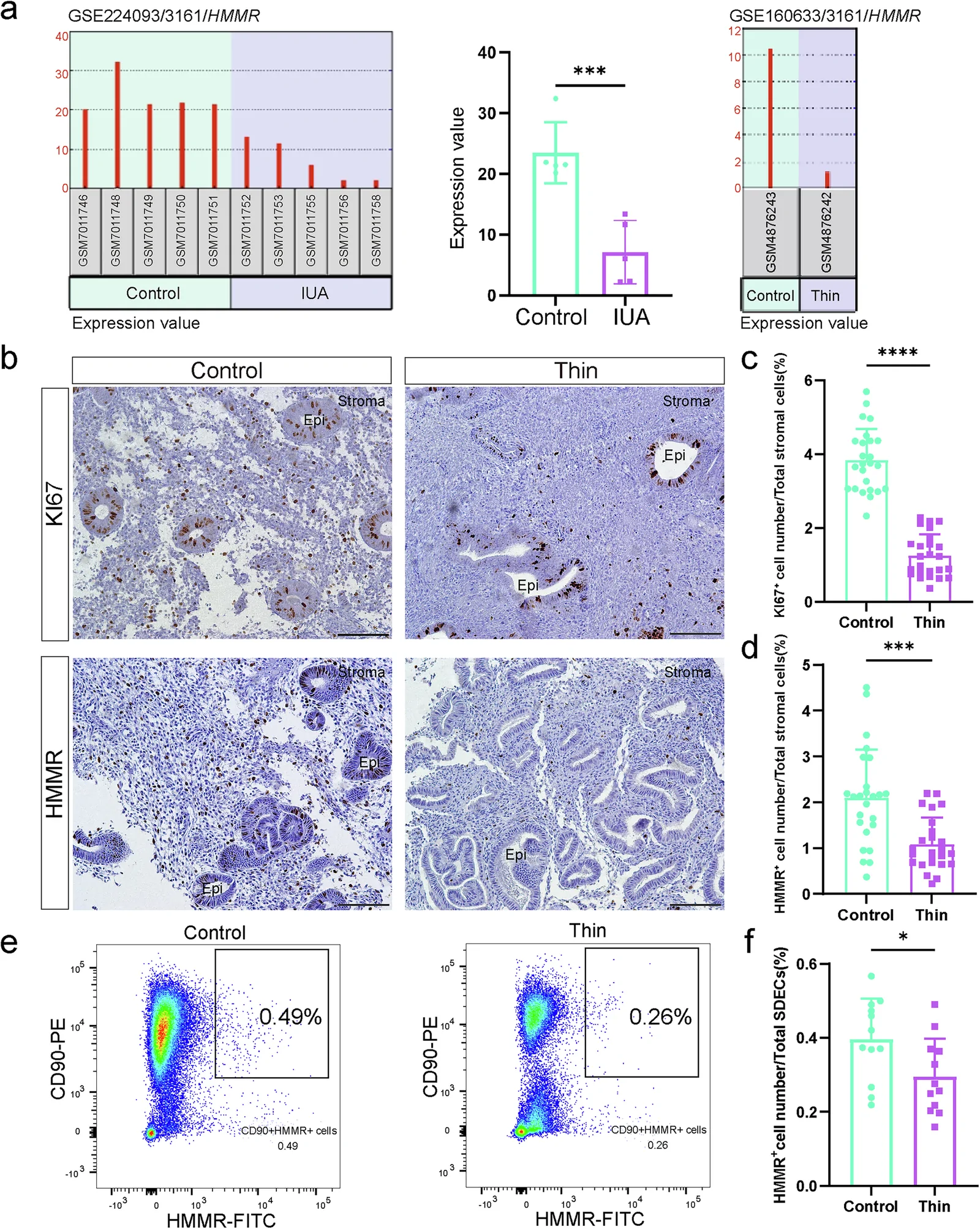
Significant reduction in the number of HMMR^+^ stromal cells in the thin endometrium. **a** Transcriptional datasets (Series: GSE224093 for IUA and GSE160633 for thin endometrium) were used to validate the specific low expression of *HMMR* in endometria with an impaired proliferative state. **b** Immunohistochemical staining of Ki67 (*n* = 25 per group) and HMMR (*n* = 25 per group) in thin endometrium (Thin) and normal endometrium (Control). Scale bars, 100 μm. **c**, **d** Quantification of Ki67^+^ cells and HMMR^+^ stromal cells in the thin endometrium compared with the normal endometrium. **e**, **f** Flow cytometric analysis of the proportions of HMMR^+^ stromal cells in the normal endometrium and thin endometrium (*n* = 12 per group). In **a**, **f**, each data point represents a separate case. In **c**, **d**, each data point represents the average of 5 fields of view from one case. Student’s *t*-tests were used for the statistical analysis. Data are expressed as mean ± SD. *P < 0.05, ** *P* < 0.01, ***P < 0.001, ****P < 0.0001; ns not significant, unpaired *t*-test.

Given the critical role of HMMR^+^ stromal cells in endometrial development and growth, we investigated whether impaired endometrial proliferation was associated with HMMR^+^ stromal cells. We observed that *HMMR* expression was significantly lower in patients with intrauterine adhesion (IUA) or a thin endometrium than in healthy controls according to known databases (Fig. 7a). To experimentally validate these observations, we performed immunohistochemical staining for Ki67 and HMMR in endometrial tissues from patients with thin endometrium and normal controls (Fig. 7b). Our results revealed that compared with that in controls (3.83% ± 0.86%, *P* < 0.0001), the Ki67 expression in the endometrial stromal cells derived from patients with thin endometrium (1.25% ± 0.58%) was significantly decreased (Fig. 7c). The proportion of HMMR^+^ stromal cells in the endometrial stromal cell fraction of the thin endometrium (0.89% ± 0.26%) was also markedly lower than that in the control endometrium (2.62% ± 1.10%, *P* < 0.001) (Fig. 7d). Furthermore, when we conducted a flow cytometric analysis of fresh endometrial tissues from patients with thin endometrium and controls, we ascertained that the proportion of HMMR^+^ stromal cells in the endometrium of patients with thin endometrium (0.26% ± 0.11%) was significantly lower than that in the controls (0.48% ± 0.02%, *P* < 0.05) (Fig. 7e, f). These data suggest that impaired endometrial proliferation in thin endometrium is strongly correlated with a reduction in the number of HMMR^+^ stromal cells.

## Discussion

The human uterine fundus, corpus, and cervix develop from the midline uterovaginal canal formed by the extensively fused bilateral Müllerian ducts^27,28^. Previous morphology and immunochemistry studies have reported that rodent and human uterine mesenchyme play essential roles in the formation of the endometrium, its differentiation into myometrial smooth muscle, and the invagination and branching of the uterine columnar epithelium^29,30^. However, the specific attributes of human uterine mesenchymal stem/progenitor cells and their underlying molecular pathways are poorly understood. In this study, we identified a population of fetal-enriched HMMR^+^ stromal cells during uterine development and during the adult menstrual cycle through single-cell and spatial transcriptomic analyses. These cells exhibited higher proliferation rates and a significant capability to differentiate into endometrial stromal cells and myometrial SMA^+^ SMCs both in vitro and in vivo. We further demonstrated that HMMR^+^ stromal cells supported epithelial gland branching and growth in the organoids through PTN signaling. In addition, HMMR^+^ stromal cells were rarely observed in the thin endometrium.

The human uterine mesenchyme originates from Müllerian mesenchymal cells that surround primitive epithelial cells and then differentiate into the uterine endometrial stroma and myometrium^31^. During the second trimester (GW14–24), these mesenchymal cells proliferate, driving uterine growth and forming three specific cell types: epithelial cells, stromal cells, and muscle cells^21^. In this study, we identified HMMR^+^ stromal cells as the progenitor-like cells critical for uterine mesenchymal development and endometrial repair during the menstrual cycle. First, these cells highly expressed proliferation-related genes, such as *Ki67*, *PCNA*, *TOP2A*, *CENPF*, and *Ube2c*, which aligns with recent findings in HMMR^+^ adipose stem cells^32^. HMMR^+^ stromal cells were predominantly in the G2/M phase and in mitosis, which is likely due to the role of HMMR in regulating spindle microtubule assembly, stability, and positioning^33,34^. Second, HMMR^+^ stromal cells exhibited an MSC phenotype and formed more CFUs in culture than HMMR^–^ stromal cells. More importantly, after in vitro induction, these differentiated HMMR^+^ stromal cells exhibited mature stromal function with high SFRP4 expression and PRL secretion^17^. After transplantation under the kidney capsules of NCG mice, these HMMR^+^ stromal cells differentiated into human endometrial mesenchymal-like tissue. These findings suggest that human HMMR^+^ stromal cells possess the potential to differentiate into various stromal cell subpopulations. Third, human endometrial HMMR^+^ stromal cells were more abundant in the fetal uterine stroma but less abundant in the adult stroma and rarely detectable in the thin endometrium. Future studies employing uterine HMMR^+^ stromal cell lineage tracing could further elucidate the role of these cells in organogenesis and cyclical endometrial regeneration.

In humans, the myometrium develops from uterine mesenchymal precursor cells surrounding the primitive endometrium^35–37^. At GW14, two layers of uterine mesenchymal cells separated by rich vascular vessels are specified. At GW18, spindle-shaped cells that resemble immature SMCs containing few cytoplasmic myofilaments appear in the outer layer of the uterus, and the expression of α-SMA becomes prominent in the uterine corpus^11,15^. Until GW20–22, the human uterine myometrium with two smooth muscle layers is well established. However, the basic cell lineage specification that governs the origin of endometrial stromal cells and SMCs has not been identified. The PV cells have been proposed as a niche for multipotent cells^38^, including CD34^+^ adventitial cells in large vessels^20^, SUSD2^+^ cells in the tunica media^39^, and CD146^+^/PDGFRb^+^ pericytes in endometrial capillaries and microvessels^20^. These cells exhibit MSC properties and, upon induction, differentiate into endometrial stroma-like cells to support cyclic regeneration^20,39^. Other candidate endometrial progenitor cells, such as LGR5^+^ cells and side population (SP) cells, remain incompletely characterized in terms of their biomarkers and localization^40,41^. Herein, we show that HMMR^+^ stromal cells are highly enriched in the fetal uterus (GW12–24), express proliferation and spindle-assembly genes (e.g., *TOP2A* and *CENPF*), and accumulate in the G2/M phase — features consistent with a transient fetal progenitor-like state rather than a quiescent adult stem cell phenotype. HMMR^+^ stromal cells possess dual-lineage potential and can differentiate into endometrial stroma and myometrial smooth muscle while supporting epithelial gland development both in vitro and in vivo. These findings suggest that this cell subset may play a unique functional role in maintaining uterine homeostasis and regeneration.

Decades of investigations using tissue recombination and developmental models have established that uterine epithelial morphogenesis, lineage specification, and gland formation are instructed by stromal signals^9,26^. Multi-omic atlases and organoid models have extended this view, revealing that adult endometrial regeneration depends on compartmentalized stromal–epithelial crosstalk mediated by niche factors such as stromal-derived FGF10, FGF2, and WNT agonists (e.g., R-spondin-3)^42,43^. However, the embryonic origin of these signaling hubs remains unknown. High-resolution spatial transcriptomics has revealed that epithelial fate is shaped by spatially restricted signaling gradients across the endometrium^12,44^. In adult regeneration, canonical WNT activity is enriched at the proliferative luminal surface, whereas gland-associated programs align with stromal non-canonical WNT cues and epithelial glandular identity factors such as FOXA2^44^. Secretory differentiation further depends on NOTCH activation within glands and local WNT inhibition (e.g., stromal DKK1)^12^. By profiling the human fetal uterus at single-cell resolution, we identified HMMR^+^ stromal cells as fetal-enriched multipotent progenitor-like cells. We propose that PTN signaling in these cells supports the paracrine instruction required for epithelial invagination and may establish stromal–epithelial networks that persist into adulthood.

Adult endometrial function — including implantation window establishment and cyclical decidualization — requires tightly synchronized communication between stromal and epithelial cells^44^. Disruption of the developmental programs that establish these interactions may compromise tissue architecture, regeneration, and receptivity. In our thin endometrium cohort, depletion of HMMR^+^ stromal cells is consistent with the possibility that key features of adult spatial signaling capacity are imprinted during fetal development. Collectively, our fetal framework bridges classic developmental biology with adult high-resolution atlases and provides new insights into how abnormalities in stromal–epithelial interactions may contribute to adult endometrial pathologies.

Owing to the scarcity of fetal samples and associated ethical considerations, our study has the following limitations. First, the rarity of fetal tissue restricted our fetal uterine sample size. Second, the sorted cells used in our biological functional assays were derived from adult rather than fetal uterine endometrial tissues. Thus, while our data suggest progenitor-like properties, they do not establish in vivo lineage relationships. Third, we did not perform in vivo lineage tracing of HMMR^+^ stromal cells, which is necessary to definitively determine their fate and contribution to uterine development.

In conclusion, primitive human HMMR^+^ stromal cells, like the multiple stromal cells, can differentiate into endometrial stromal cells and myometrial SMCs and promote endometrial epithelial cell development by inducing PTN signaling. Their deficiency impairs endometrial stromal and epithelial cell proliferation, leading to a thin endometrium. These findings contribute to a comprehensive understanding of the cellular composition and development of the fetal uterus, potentially facilitating the diagnosis and treatment of uterine disorders.

## Materials and methods

### Collection of human tissues

Human uterine samples were collected, and procedures were conducted at the Nanjing Drum Tower Hospital, with approval from the hospital’s ethics committee (Approval No. 2021-131-02). Human fetal uterine corpus tissues were obtained in strict accordance with legal and institutional ethical regulations, following the acquisition of informed consent from the donors. The fetal uterine tissues were collected within 6 h post abortion under sterile conditions. The adult full-thickness uterine corpus samples were collected from patients who underwent hysterectomy for cervical cancer during two menstrual cycle stages (proliferative and secretory phases). Tissues were excised from macroscopically normal areas of the uterus using a scalpel. These samples were used for single-cell transcriptomics, spatial transcriptomics, immunohistochemistry, and immunofluorescence validation.

Adult endometrial samples were collected using endometrial biopsy curettes from reproductive-age women (18–35 years), including patients with thin endometrium (mid-luteal phase endometrial thickness < 7 mm by ultrasound) and age-matched healthy controls (mid-luteal phase endometrial thickness ≥ 8 mm and < 14 mm by ultrasound). The inclusion criteria included a body mass index (BMI) between 18 and 25, a regular menstrual cycle (25–37 days with 3–5 days of menstruation), normal ovarian function (AMH ≥ 1 ng/mL), no hormonal treatment during the collection month, and sample collection during the proliferative phase. All participants underwent endometrial scratching, exhibited normal ovulatory function, and had no evidence of reproductive system malignancies, including cervical or ovarian cancer. These samples were used for fluorescence-activated cell sorting (FACS) of HMMR^+^ and HMMR⁻ stromal cells, as well as for immunohistochemistry and flow cytometry for thin endometrial pathological analyses.

The samples were immediately placed in PBS on ice and transported to the laboratory for subsequent experiments. Written informed consent was obtained from all participants.

### scRNA-seq and analysis

In this study, full-thickness uterine corpus tissue samples were collected at various developmental stages, including the fetal stages of GW12, GW16, GW20, GW22, GW23, and GW24, as well as adult samples from the proliferative and secretory phases of the menstrual cycle. Fetal samples were obtained by isolating the uterus and bilateral adnexa from aborted fetuses using sterile instruments under a laminar flow hood. The tissues were washed twice with sterile PBS and then separated at the junction of the uterus and fallopian tubes using sterile ophthalmic scissors. For adult samples, a segment of full-thickness uterine tissue was excised from a total hysterectomy specimen using a surgical blade. Sampling was performed carefully to avoid ovarian cell contamination. All the samples were preserved in tissue preservation solution and transported at low temperatures to Singleron Biotechnologies in Nanjing for single-cell sequencing processing. First, the tissues were dissociated into single-cell suspensions using mechanical methods and SCelLiVe® dissociation solution. The single-cell suspensions were processed with the Singleron Matrix® automated single-cell sequencing library construction system, which dispersed the cells into the high-density microwell array of the SCOPE-chip® microfluidic chip. This system automated the processes of cell separation, cell lysis, and mRNA capture. The constructed libraries were then sent to a high-throughput sequencing platform for sequencing. The sequencing data underwent quality control, read alignment, and cell cluster identification, resulting in the generation of gene expression matrices. We subsequently used the Seurat package to further filter cells, retaining those with gene expression levels between 200 and 2500 and a mitochondrial gene expression proportion below 40%. The filtered data were then normalized and subjected to dimensionality reduction using principal component analysis (PCA), with the first 20 principal components selected for clustering analysis. We employed Harmony to integrate all sample data and correct batch effects. Cell clustering was performed using the graph-based Louvain algorithm, and the distribution of different cell types was visualized with the UMAP method, followed by annotation with known cell type markers. Differentially expressed genes between cell types were identified using the FindMarkers function, followed by functional enrichment analysis. Gene expression visualization was performed using the Nebulosa package^45^. Additionally, Cytotrace was utilized to assess cell developmental states and infer differentiation trajectories. To elucidate cellular differentiation trajectories and key developmental transitions, we adopted a standardized sampling strategy, in which equal numbers of cells from each group were extracted, followed by pseudotime analysis using Monocle2.

### Spatial transcriptomics data processing and analysis

Human uterine tissue samples were sent to GENECHEM (Shanghai, China) for spatial transcriptome profiling on the 10X Genomics Visium Spatial Gene Expression (second-generation) platform following the manufacturer’s instructions. Fresh tissue sections (10 μm) were mounted on Visium slides, fixed, and subjected to H&E staining for histological assessment. After tissue permeabilization, spatially barcoded cDNA libraries were generated and sequenced on an Illumina NovaSeq 6000 platform. Raw sequencing data were processed using the Space Ranger pipeline (v2.0, 10X Genomics, Pleasanton, CA, USA) for alignment to the human reference genome (GRCh38), UMI counting, and spatial barcode assignment. Low-quality spots (fewer than 500 detected genes or those with high mitochondrial content) were excluded. Downstream analyses, including normalization, dimensionality reduction, clustering, and visualization, were performed in RStudio using Seurat (v4.3.0) and R (v4.3.1). We used RCTD to deconvolve the spatial transcriptomic spots, referencing cell type annotations from our scRNA-seq data of human uterine tissues. This approach resulted in the mapping of distinct cell identities onto the spatial array, and the inferred compositions were visualized as color-coded proportions for direct comparison with tissue histology.

### Immunohistochemistry and immunofluorescence

Tissues, including full-thickness human uterine samples and mouse kidney samples, were snap-frozen in liquid nitrogen and then embedded in a Tissue-Tek OCT compound for further analysis (Sakura Finetech, Torrance, CA, USA). Serial frozen sections were then cut at 5–10 µm thickness using a Leica cryostat (Leica Microsystems, Germany). The frozen sections were air-dried for 10 min at room temperature. The tissues were then fixed with 4% paraformaldehyde for 5 min, followed by permeabilization with PBS containing 0.1% Triton X-100 for 10 min. The tissue sections were blocked with 10% goat serum for 40 min, followed by overnight incubation at 4 °C with prediluted primary antibodies in antibody dilution medium (Invitrogen, Carlsbad, CA, USA; details provided in Supplementary Table S1). After the sections were washed with TBST, they were incubated with appropriate secondary antibodies at room temperature for 60 min, followed by nuclear staining with DAPI (Invitrogen), and then the expression of various molecules was analyzed using a fluorescence confocal microscope (Leica, Wetzlar, Germany) or a fluorescence microscope (Leica).

For immunohistochemistry staining, tissues, including human endometrium samples and mouse kidney samples, were fixed in 10% neutral formaldehyde for 24 h and then embedded perpendicularly in paraffin. Transverse sections were cut to a thickness of 3 μm. The tissue sections were incubated overnight at 4 °C with primary antibodies (details are provided in Supplementary Table S1). The sections were subsequently incubated with goat anti-rabbit IgG and an avidin-biotin complex (Boster, Wuhan, China) for 1 h each at room temperature. Finally, the sections were stained with 3,3-diaminobenzidine and counterstained with hematoxylin.

### Cell sorting and culture

Human endometrial tissues from adult women were cut into small pieces and digested in PBS (Gibco, Grand Island, NY, USA) supplemented with 1 mg/mL type I collagenase (Sigma, St. Louis, MO, USA) at 37 °C for 30–45 min. The digested cell suspension was passed through a 100 μm cell strainer (Corning, Falcon, NY, USA), mixed with 1× red blood cell lysis buffer (Beyotime, Shanghai, China), and incubated at 4 °C for 15 min. The cells were filtered through a 40 μm cell strainer (Corning) and centrifuged. The cell pellets were then resuspended in 1% FBS/PBS and incubated with an antibody cocktail containing anti-CD45-APC-Cy7 (BD Biosciences, San Jose, CA, USA), anti-CD56-PE-Cy7 (BD Biosciences), anti-CD144-PerCP-Cy5.5 (BD Biosciences), anti-CD90-PE (BD Biosciences), and anti-HMMR-FITC (Proteintech, Wuhan, China) at 4 °C for 40 min. After being washed with 1% FBS/PBS, CD45^–^CD56^–^CD144^–^CD90^+^HMMR^+^ and CD45^–^CD56^–^CD144^–^CD90^+^HMMR^–^ endometrial stromal cells were sorted by a BD FACSAria™ III Cell Sorter (BD Biosciences, San Jose, CA, USA). To isolate endometrial stromal cells, we excluded CD45^+^ immune cells, CD56^+^ myogenic cells, and CD144^+^ endothelial/epithelial cells, and positively selected for CD90^+^ stromal cells. These cells were subsequently further subdivided into CD90^+^HMMR^+^ and CD90^+^HMMR^–^ populations. Viable cells were identified as negative for DAPI staining. CD90 fluorescence minus one (FMO) staining tubes and HMMR FMO staining tubes were utilized to evaluate antibody specificity and background fluorescence. The sorted HMMR^+^ and HMMR^–^ stromal cells were cultured in a 37 °C, 5% CO_2_ incubator with DMEM/F12 (Gibco) supplemented with 10% FBS (Gibco), 1% penicillin‒streptomycin (Gibco), and 5 ng/mL bFGF (Gibco) and passaged upon reaching 80%–90% confluence.

### Flow cytometry analysis

The surface antigens of cultured HMMR^+^ and HMMR^–^ stromal cells (at passage 3) were analyzed using flow cytometry (BD Biosciences). Cells were initially digested, resuspended in PBS, and incubated for 40 min at room temperature with anti-HMMR-FITC (Proteintech), anti-CD13-PE (eBioscience, San Diego, CA, USA), anti-CD29-FITC (eBioscience), anti-CD34-PE (Multisciences, Hangzhou, China), anti-CD44-FITC (BD Pharmingen, San Jose, CA, USA), anti-CD45-FITC (eBioscience), anti-CD73-FITC (eBioscience), anti-CD90-FITC (eBioscience), anti-CD105-PE (eBioscience), anti-CD144 (BD Pharmingen) and anti-HLA-DR-APC (Multisciences) while protected from light. After incubation, the cells were centrifuged, washed, and resuspended in 1% FBS/PBS for analysis.

### qPCR

Total RNA was extracted using TRIzol reagent (Invitrogen). RNA purity and concentration were assessed by spectrophotometry. cDNA was synthesized from 1 μg of purified RNA using a HiScript III RT SuperMix kit (Vazyme, Nanjing, China). The primer sequences used are listed in Supplementary Table S2. Each sample was analyzed in biological triplicate, and the relative expression of each mRNA was normalized to that of the respective control (*GAPDH* or *18S*).

### Western blotting

Cells were washed with PBS, treated with RIPA lysis buffer (Epizyme, Shanghai, China) supplemented with protease inhibitors (Beyotime), and then scraped on ice. After quantifying protein concentration using a BCA assay (Beyotime), an equivalent volume of 40 μg of total protein was used for western blotting. Proteins separated by SDS-PAGE were then transferred to a PVDF membrane (Millipore, Bedford, MA, USA). Blocking was performed with 5% skim milk at room temperature for 1.5 h, followed by overnight incubation at 4 °C with primary antibodies (HMMR, 1:1000; α-SMA, 1:8000). Goat anti-rabbit IgG (Abways, Shanghai, China) was used as the secondary antibody at room temperature for 1 h. The bands were visualized using an enhanced chemiluminescence (ECL) kit (Beyotime).

### Cell proliferation assay

HMMR^+^ and HMMR^–^ stromal cells were suspended and seeded onto 96-well plates at 1 × 10^3^ cells per well. CCK-8 reagent (10 μL; Fdbio Science, Hangzhou, China) was added to each group after 24 h, 48 h, 72 h, 96 h, 120 h, 144 h, and 168 h of culture. Following a 2-h incubation at 37 °C, cell proliferation was assessed by measuring the absorbance at 450 nm using a microplate reader (Thermo, MA, USA).

### Colony formation assay

HMMR^+^ and HMMR^–^ stromal cells were seeded onto 6-well plates (1 × 10^3^ cells per well) and incubated at 37 °C and 5% CO_2_ for 2 weeks. The cells were fixed with 4% paraformaldehyde for 20 min at room temperature and stained with 0.1% crystal violet staining solution (Servicebio, Wuhan, China). The number of clusters with more than 50 cells was counted after the cells were washed with PBS.

### Differentiation into endometrial stromal subpopulations

HMMR^+^ and HMMR^–^ stromal cells were initially cultured in 6-well plates (Corning) at a density of 1 × 10^5^ cells per well and serum-starved overnight. They were subsequently cultured in low-serum medium (DMEM/F12 with 2% FBS) supplemented with 0.5 mM 8-Br-CAMP (MedChem Express, Monmouth Junction, NJ, USA), 10 nM 17β-estradiol (E2) (Sigma), 1 μM 17-OH-Progesterone (P4) (Sigma), and 1 nM BMP2 (PeproTech, Rocky Hill, NJ, USA). The culture medium was changed every three days. After 21 days, the concentration of PRL in the collected medium was measured using an ELISA kit (Cloud-Clone Corp, Wuhan, China) according to the manufacturer’s instructions, and the absorbance was read on a Thermo microplate reader. Intracellular levels of SFRP4 were quantified using qPCR.

### Lentiviral transduction of HMMR^+/–^ stromal cells

HMMR^+^ and HMMR⁻ stromal cells were isolated from human uterine tissues as described above and cultured in an appropriate growth medium. Cells were seeded at a density of 1 × 10^5^ cells per well in 6-well plates and allowed to adhere overnight. Recombinant mCherry-expressing lentivirus (GENECHEM, Shanghai, China) and infection solution A were added at a multiplicity of infection (MOI) of 50. After 12–16 h, the medium was replaced with fresh growth medium. Transduction efficiency was evaluated at 72 h post infection by fluorescence microscopy and flow cytometry. mCherry^+^ cells were collected and used directly for the subsequent in vivo transplantation experiments.

### Xenotransplantation into the mouse kidney capsule

The animal experiments were approved by the Animal Research Ethics Review Committee of the Nanjing Drum Tower Hospital (Approval No. 2024AE01024). Six-week-old female NCG mice (GemPharmatech, Nanjing, China) were maintained under SPF conditions at the Nanjing Drum Tower Hospital Laboratory Animal Center to allow them to acclimatize to their new environment. A detailed procedure for xenografting into a mouse kidney model using 6–8-week-old female NCG mice was described in our previous study^20^. In brief, NCG mice were initially ovariectomized and implanted with two E_2_ pellets (1.7 mg of E2 per pellet; E-121; Innovative Research of America, Sarasota, FL, USA) before transplantation, and then injected with 20 μL of a 1:1 mixture of DMEM/F12 and Matrigel (Corning, NY, USA) containing 1 × 10^6^ cells under the capsule of each kidney using an insulin needle. In the control group, SDECs not subjected to viral transduction were implanted under the kidney capsule (*n* = 3 mice, totaling six kidneys analyzed). Equivalent numbers of HMMR^+^ and HMMR⁻ stromal cells carrying an mCherry lentiviral label were transplanted under the kidney capsule into mice of separate groups (*n* = 3 mice per group). After two months, the graft-bearing kidneys were excised and analyzed through photography, H&E staining, immunohistochemistry, and immunofluorescence staining.

### Differentiation into SMCs

HMMR^+^ and HMMR^–^ stromal cells were initially cultured in 6-well plates (Corning) at a density of 1 × 10^5^ cells per well. The cells were subsequently induced to differentiate into SMCs by culturing them in serum-free medium (DMEM/F12) supplemented with 2.5 ng/mL TGF-β (Novoprotein, Shanghai, China)^22^. The medium was replaced every two days, and the cells were maintained in culture for a total duration of eight days. Following the induction period, smooth muscle differentiation markers (α-SMA) were detected using immunofluorescence staining and western blot analysis, and the gene expression levels of smooth muscle-related markers (α-SMA, CNN1, and SM22-α) were quantified by qPCR. Uninduced HMMR^+^ and HMMR⁻ stromal cells cultured in standard medium served as controls.

### Transfection of siRNA

PTN siRNA and negative control siRNA were purchased from General Biol (Chuzhou, China). For transfection, HMMR^+^ stromal cells were separately cultured in a humidified incubator at 37 °C with 5% CO_2_ in DMEM/F12 medium until they reached ~70%–80% confluence. siRNA (at a final concentration of 20 nM) was diluted in 500 μL of Opti-MEM (Gibco), to which 7 μL of Lipofectamine 3000 (Invitrogen) was added. The mixture was incubated for 20 min at room temperature prior to being added to the cells. The cells were incubated with the siRNA mixture for 6–8 h in a humidified incubator at 37 °C. After 48 h of incubation, the cells were harvested for RNA and protein extraction. PTN knockdown efficiency was then assessed using qPCR for RNA analysis and western blotting for protein analysis.

### Human endometrial epithelial organoids co-cultured with stromal cells

First, the human endometrial tissue was mechanically minced and digested with 1 mg/mL collagenase I (Sigma) at 37 °C for 20–30 min. After digestion, the resulting cell suspension was collected, and red blood cells were removed using Red Blood Cell Lysis Buffer (Beyotime). The supernatant was then neutralized, and stromal cells were isolated using a 70 μm cell strainer (Corning). Epithelial cells were obtained by backwashing and subjected to further digestion with TrypLE (Thermo Fisher Scientific) at 37 °C for 20 min to generate a single-cell suspension. The cell suspension with 1 × 10^4^ epithelial cells and 1 × 10^4^ stromal cells (either HMMR^+^, HMMR^−^, or si-PTN) was mixed with chilled Matrigel (Corning) at a 1:2 ratio (v:v). A 30 μL droplet of the Matrigel-cell mixture was plated into 48-well plates (Corning), solidified at 37 °C for 10 min, and overlaid with 200 μL of endometrial organoid expansion medium (ExM).

The ExM was derived from a previous study and consisted of N2 supplement (Life Technologies, Carlsbad, CA, USA), B27 supplement (Life Technologies), 100 μg/mL primoccin (InvivoGen, Toulouse, France), 1.25 mM N-acetyl-L-cysteine (Sigma), 50 ng/mL EGF (Peprotech), 100 ng/mL Noggin (Peprotech), 500 ng/mL R-spondin-1 (Peprotech), 2 mM L-glutamine (Life Technologies), 100 ng/mL FGF10 (Peprotech), 50 ng/mL HGF (Peprotech), 500 nM ALK-4, -5, -7 inhibitor, A83-01 (System Biosciences, Palo Alto, CA, USA), and 10 nM nicotinamide (Sigma) in advanced DMEM/F12 medium (Gibco). Organoids were cultured at 37 °C in 5% CO_2_, with the medium changed every 2–3 days. After 14 days of culture, the organoids were photographed and counted.

### Endometrial organoid scRNA-seq

Monocultured endometrial organoids or endometrial organoids co-cultured with HMMR^+^ stromal cells were maintained in ExM at 37 °C with 5% CO_2_ for 14 days. Organoids were subsequently harvested and processed for scRNA-seq by Singleron Biotechnologies (Nanjing, China).

### Statistical analyses

Statistical analyses were conducted using *t*-test for two-group comparisons and ANOVA for multiple-group comparisons. Data are represented as mean ± SD. Statistical significance was assessed using GraphPad Prism version 9 (GraphPad Software, La Jolla, CA, USA), with a *P*-value of < 0.05 considered statistically significant.

## Supplementary information

Supplementary information

## Data Availability

The raw sequence data reported in this paper have been deposited in the Genome Sequence Archive (Genomics, Proteomics & Bioinformatics 2025) in the National Genomics Data Center (Nucleic Acids Res 2025), China National Center for Bioinformation/Beijing Institute of Genomics, Chinese Academy of Sciences (GSA-Human: HRA019335 and HRA009207) that are publicly accessible at https://ngdc.cncb.ac.cn/gsa-human^46,47^. This paper does not report original code. Any additional information required to reanalyze the data reported in this paper is available from the lead contact upon request.

## References

[1] Yu, D., Wong, Y. M., Cheong, Y., Xia, E. & Li, T. C. Asherman syndrome-one century later. *Fertil. Steril.* **89**, 759–779 (2008). 18406834 10.1016/j.fertnstert.2008.02.096

[2] Makiyan, Z. Studies of gonadal sex differentiation. *Organogenesis* **12**, 42–51 (2016). 26950283 10.1080/15476278.2016.1145318 PMC4882125

[3] Roly, Z. Y. et al. The cell biology and molecular genetics of Müllerian duct development. *Wiley Interdiscip. Rev. Dev. Biol.* **7**, e310 (2018). 29350886 10.1002/wdev.310

[4] Chandler, T. M., Machan, L. S., Cooperberg, P. L., Harris, A. C. & Chang, S. D. Mullerian duct anomalies: from diagnosis to intervention. *Br. J. Radiol.* **82**, 1034–1042 (2009). 19433480 10.1259/bjr/99354802 PMC3473390

[5] Lorenzi, V. et al. Spatiotemporal cellular map of the developing human reproductive tract. *Nature* **650**, 428–437 (2026). 41407855 10.1038/s41586-025-09875-2 PMC12893920

[6] Cao, J. et al. The single-cell transcriptional landscape of mammalian organogenesis. *Nature* **566**, 496–502 (2019). 30787437 10.1038/s41586-019-0969-x PMC6434952

[7] Han, X. et al. Mapping the mouse cell atlas by Microwell-seq. *Cell* **172**, 1091–1107.e17 (2018). 29474909 10.1016/j.cell.2018.02.001

[8] Tabula Muris Consortium et al. Single-cell transcriptomics of 20 mouse organs creates a Tabula Muris. *Nature* **562**, 367–372 (2018). 30283141 10.1038/s41586-018-0590-4 PMC6642641

[9] Cunha, G. R. et al. Molecular mechanisms of development of the human fetal female reproductive tract. *Differentiation* **97**, 54–72 (2017). 29053991 10.1016/j.diff.2017.07.003 PMC5940488

[10] Koff, A. K. Development of the vagina in the human fetus. *Contrib. Embryol.* **24**, 59–91 (1933). 12332362

[11] Robboy, S. J., Kurita, T., Baskin, L. & Cunha, G. R. New insights into human female reproductive tract development. *Differentiation* **97**, 9–22 (2017). 28918284 10.1016/j.diff.2017.08.002 PMC5712241

[12] Garcia-Alonso, L. et al. Mapping the temporal and spatial dynamics of the human endometrium in vivo and in vitro. *Nat. Genet.* **53**, 1698–1711 (2021). 34857954 10.1038/s41588-021-00972-2 PMC8648563

[13] Szamborski, J. & Laskowska, H. Some observations on the developmental histology of the human foetal uterus. *Biol. Neonat.* **13**, 298–314 (1968). 4186110 10.1159/000240156

[14] Cunha, G. R. et al. Development of the human female reproductive tract. *Differentiation* **103**, 46–65 (2018). 30236463 10.1016/j.diff.2018.09.001 PMC6234064

[15] Fritsch, H., Hoermann, R., Bitsche, M., Pechriggl, E. & Reich, O. Development of epithelial and mesenchymal regionalization of the human fetal utero-vaginal anlagen. *J. Anat.* **222**, 462–472 (2013). 23406280 10.1111/joa.12029 PMC3610038

[16] Bao, H. et al. PR-SET7 epigenetically restrains uterine interferon response and cell death governing proper postnatal stromal development. *Nat. Commun.* **15**, 4920 (2024). 38858353 10.1038/s41467-024-49342-6 PMC11164956

[17] Wu, B. et al. SFRP4(+) stromal cell subpopulation with IGF1 signaling in human endometrial regeneration. *Cell Discov.* **8**, 95 (2022). 36163341 10.1038/s41421-022-00438-7 PMC9512788

[18] Hanaki, S. & Shimada, M. Impact of FKBP52 on cell proliferation and hormone-dependent cancers. *Cancer Sci.* **114**, 2729–2738 (2023). 37026526 10.1111/cas.15811 PMC10323096

[19] Yi, M., Negishi, M. & Lee, S. J. Estrogen sulfotransferase (SULT1E1): its molecular regulation, polymorphisms, and clinical perspectives. *J. Pers. Med.* **11**, 194 (2021). 33799763 10.3390/jpm11030194 PMC8001535

[20] Zhu, X. et al. Human endometrial perivascular stem cells exhibit a limited potential to regenerate endometrium after xenotransplantation. *Hum. Reprod.* **36**, 145–159 (2021). 33283858 10.1093/humrep/deaa261

[21] Habiba, M., Heyn, R., Bianchi, P., Brosens, I. & Benagiano, G. The development of the human uterus: morphogenesis to menarche. *Hum. Reprod. Update* **27**, 1–26 (2021). 33395479 10.1093/humupd/dmaa036

[22] Ross, J. J. et al. Cytokine-induced differentiation of multipotent adult progenitor cells into functional smooth muscle cells. *J. Clin. Invest.* **116**, 3139–3149 (2006). 17099777 10.1172/JCI28184 PMC1635164

[23] Zhang, L. et al. Creatine promotes cancer metastasis through activation of Smad2/3. *Cell Metab.* **33**, 1111–1123.e4 (2021). 33811821 10.1016/j.cmet.2021.03.009

[24] Hapangama, D. K. et al. Abnormally located SSEA1+/SOX9+ endometrial epithelial cells with a basalis-like phenotype in the eutopic functionalis layer may play a role in the pathogenesis of endometriosis. *Hum. Reprod.* **34**, 56–68 (2019). 30496412 10.1093/humrep/dey336 PMC6295963

[25] Tempest, N., Baker, A. M., Wright, N. A. & Hapangama, D. K. Does human endometrial LGR5 gene expression suggest the existence of another hormonally regulated epithelial stem cell niche. *Hum. Reprod.* **33**, 1052–1062 (2018). 29648645 10.1093/humrep/dey083 PMC5972618

[26] Kelleher, A. M., DeMayo, F. J. & Spencer, T. E. Uterine glands: developmental biology and functional roles in pregnancy. *Endocr. Rev.* **40**, 1424–1445 (2019). 31074826 10.1210/er.2018-00281 PMC6749889

[27] Kurita, T. & Nakamura, H. Embryology of the uterus. in *The Endometrium: Molecular, Cellular and Clinical Perspectives* (Aplin, J. D., Fazleabas, A. T., Glasser S. R. & Giudice, L. C. eds), CRC Press, 1–18 (2008).

[28] Kobayashi, A. & Behringer, R. R. Developmental genetics of the female reproductive tract in mammals. *Nat. Rev. Genet.* **4**, 969–980 (2003). 10.1038/nrg1225 14631357

[29] Aboseif, S., El-Sakka, A., Young, P. & Cunha, G. Mesenchymal reprogramming of adult human epithelial differentiation. *Differentiation* **65**, 113–118 (1999). 10550544 10.1046/j.1432-0436.1999.6520113.x

[30] Cunha, G. R. et al. Role of epithelial-mesenchymal interactions in the differentiation and spatial organization of visceral smooth muscle. *Epithel. Cell Biol.* **1**, 76–83 (1992). 1307941

[31] Huang, C. C., Orvis, G. D., Kwan, K. M. & Behringer, R. R. Lhx1 is required in Müllerian duct epithelium for uterine development. *Dev. Biol.* **389**, 124–136 (2014). 24560999 10.1016/j.ydbio.2014.01.025 PMC3988469

[32] Zhang, X. et al. Hmmr acts as a key regulator in the ADSCs proliferation and mitosis. *Stem Cell Rev. Rep.* **19**, 1937–1953 (2023). 37222947 10.1007/s12015-023-10563-9

[33] Chen, H., Connell, M., Mei, L., Reid, G. & Maxwell, C. A. The nonmotor adaptor HMMR dampens Eg5-mediated forces to preserve the kinetics and integrity of chromosome segregation. *Mol. Biol. Cell* **29**, 786–796 (2018). 29386294 10.1091/mbc.E17-08-0531 PMC5905292

[34] Maxwell, C. A., McCarthy, J. & Turley, E. Cell-surface and mitotic-spindle RHAMM: moonlighting or dual oncogenic functions? *J. Cell Sci.* **121**, 925–932 (2008). 18354082 10.1242/jcs.022038

[35] Konishi, I., Fujii, S., Okamura, H. & Mori, T. Development of smooth muscle in the human fetal uterus: an ultrastructural study. *J. Anat.* **139**, 239–252 (1984). 6490516 PMC1164372

[36] Kurita, T. Normal and abnormal epithelial differentiation in the female reproductive tract. *Differentiation* **82**, 117–126 (2011). 21612855 10.1016/j.diff.2011.04.008 PMC3178098

[37] Liu, S. et al. Nonsense mutation of EMX2 is potential causative for uterus didelphysis: first molecular explanation for isolated incomplete müllerian fusion. *Fertil. Steril.* **103**, 769–774.e2 (2015). 25577462 10.1016/j.fertnstert.2014.11.030

[38] Li, S. & Ding, L. Endometrial perivascular progenitor cells and uterus regeneration. *J. Pers. Med*. **11**, 477 (2021). 10.3390/jpm11060477 PMC8230145 34071743

[39] Masuda, H., Anwar, S. S., Bühring, H. J., Rao, J. R. & Gargett, C. E. A novel marker of human endometrial mesenchymal stem-like cells. *Cell Transpl.* **21**, 2201–2214 (2012). 10.3727/096368911X637362 22469435

[40] Cervelló, I. et al. Leucine-rich repeat-containing G-protein-coupled receptor 5-positive cells in the endometrial stem cell niche. *Fertil. Steril.* **107**, 510–519.e3 (2017). 27887719 10.1016/j.fertnstert.2016.10.021

[41] Kato, K. et al. Characterization of side-population cells in human normal endometrium. *Hum. Reprod.* **22**, 1214–1223 (2007). 17283036 10.1093/humrep/del514

[42] Kurita, T., Cooke, P. S. & Cunha, G. R. Epithelial–stromal tissue interaction in paramesonephric (Müllerian) epithelial differentiation. *Dev. Biol.* **240**, 194–211 (2001). 11784056 10.1006/dbio.2001.0458

[43] Turco, M. Y. et al. Long-term, hormone-responsive organoid cultures of human endometrium in a chemically defined medium. *Nat. Cell Biol.* **19**, 568–577 (2017). 28394884 10.1038/ncb3516 PMC5410172

[44] Wang, W. et al. Single-cell transcriptomic atlas of the human endometrium during the menstrual cycle. *Nat. Med.* **26**, 1644–1653 (2020). 32929266 10.1038/s41591-020-1040-z

[45] Alquicira-Hernandez, J. & Powell, J. E. Nebulosa recovers single-cell gene expression signals by kernel density estimation. *Bioinformatics* **37**, 2485–2487 (2021). 33459785 10.1093/bioinformatics/btab003

[46] CNCB–NGDC Members and Partners. Database resources of the National Genomics Data Center, China National Center for Bioinformation in 2026. *Nucleic Acids Res.* **54**, D28–D47 (2026). 10.1093/nar/gkaf1172 PMC12807729 41359036

[47] Zhang, S. et al. The GSA family in 2025: a broadened sharing platform for multi-omics and multimodal data. *Genom. Proteom. Bioinform.* **23**, qzaf072 (2025). 10.1093/gpbjnl/qzaf072 PMC12451262 40857552

