# Supplementary material for: HMMR+ stromal cells drive human uterine development
Source: Cell Discov. 2026 Sep 8;12:65. doi: 10.1038/s41421-026-00921-5 (PMC13550414; doi:10.1038/s41421-026-00921-5)
Supplement: Supplementary file 1 [file 41421_2026_921_MOESM1_ESM.pdf]

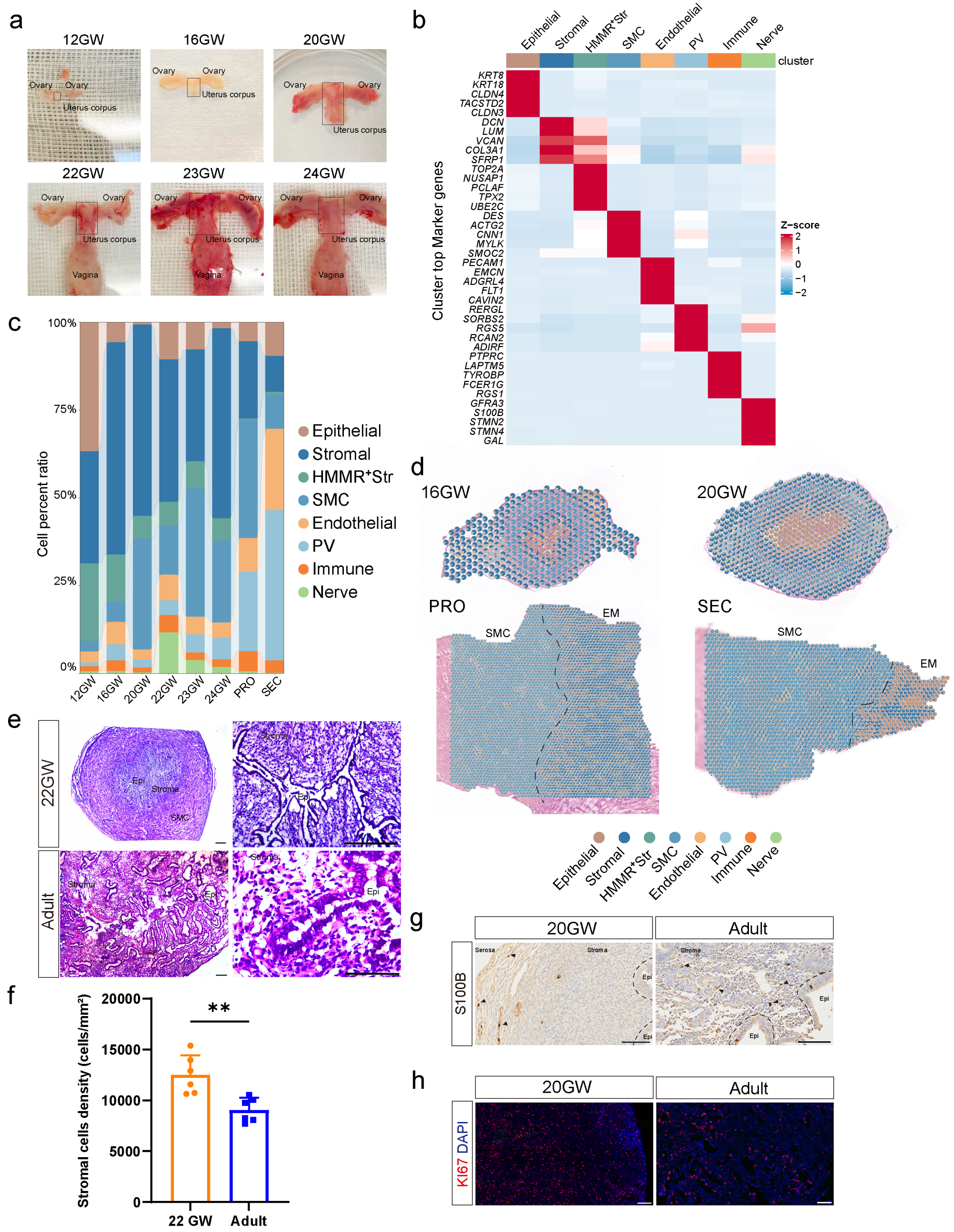

Supplementary Fig S1

Supplementary Fig S1. The gross and molecular character of human fetal and adult uteruses, related to Fig 1

(a) Schematic diagrams of uterine tissues at GW12, 16, 20, 22, 23, and 24.

(b) Heatmap showing the top 5 specifically expressed genes for each of the 8 main clusters.

(c) The proportion of each cell type in the uterus at different developmental stages.

(d) Spatial mapping of all cell types in transverse sections of the fetal uterus (GW16, GW20) and full-thickness uterine sections from proliferative (PRO) and secretory (SEC) phases, with colors representing the predicted percentage of each cell type per Visium spot based on RCTD deconvolution.

(e) Hematoxylin and eosin (H&E) staining of uteruses at GW22 and in adulthood.

(f) Quantitative analysis of Vimentin immunofluorescence staining in GW22 fetal and adult uterine sections was performed to quantify stromal cell density. The analysis revealed a higher stromal cell density in the fetal uterus relative to the adult uterus.

(g) Immunohistochemical staining of S100B in GW20 fetal and adult uterine sections, showing the distribution of

neural/immune cells. Scale bars, 100 μm.

(h) Immunofluorescent staining of KI67 (proliferating cells) in GW22 and adult uterus. Scale bars, 100 μm.

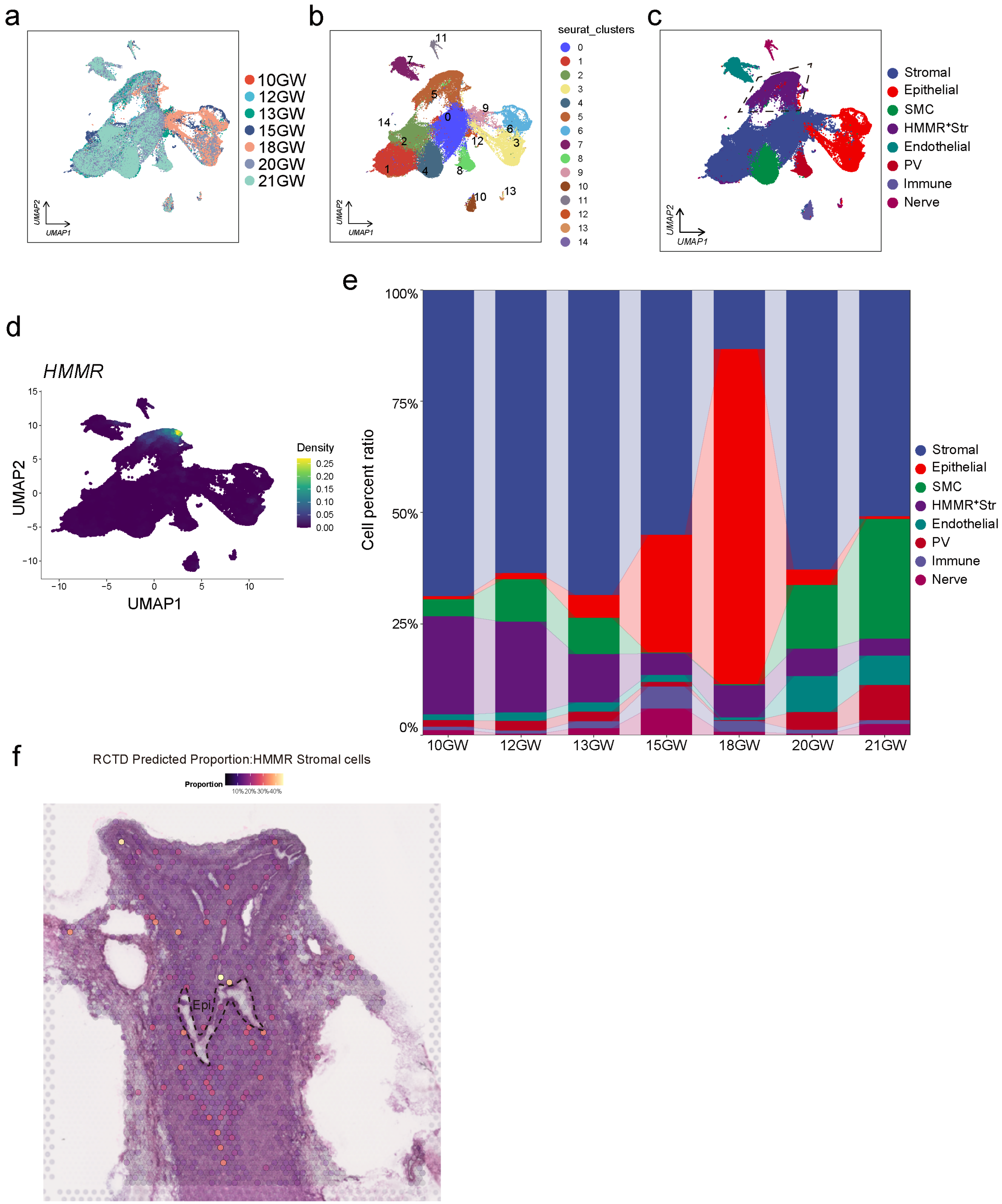

Supplementary Fig S2

Supplementary Fig S2. Independent validation of HMMR+ stromal cells using a public fetal uterine scRNA-seq and spatial transcriptomics dataset, related to Fig 1

(a) UMAP plot of all uterine cells from the public fetal scRNA-seq dataset (PMID: 41407855), colored by developmental stage. The analysis included samples from seven gestational stages: GW10, GW12, GW13, GW15, GW18, GW20, and GW21.

(b) UMAP plot colored by unsupervised clustering results, showing 15 distinct cell clusters.

(c) UMAP plot of cells from fetal full-thickness uterine samples, with the 15 unsupervised clusters merged and annotated as eight major cell types based on our data integration. HMMR+ Str, HMMR+ stromal cells; SMC, smooth muscle cells; PV, perivascular cells; Nerve, neural cells.

(d) Plot density depicted the canonical marker *HMMR* for the HMMR+ stromal cell subpopulation.

(e) The proportion of each cell type in the uterus at different developmental stages.

(f) Spatial localization of HMMR+ stromal cells (HMMR+ Str) in a sagittal section of the fetal uterus at GW18, inferred by RCTD deconvolution of Visium spatial transcriptomics data.

Color fill proportions within each Visium spot indicate the predicted abundance of each cell type.

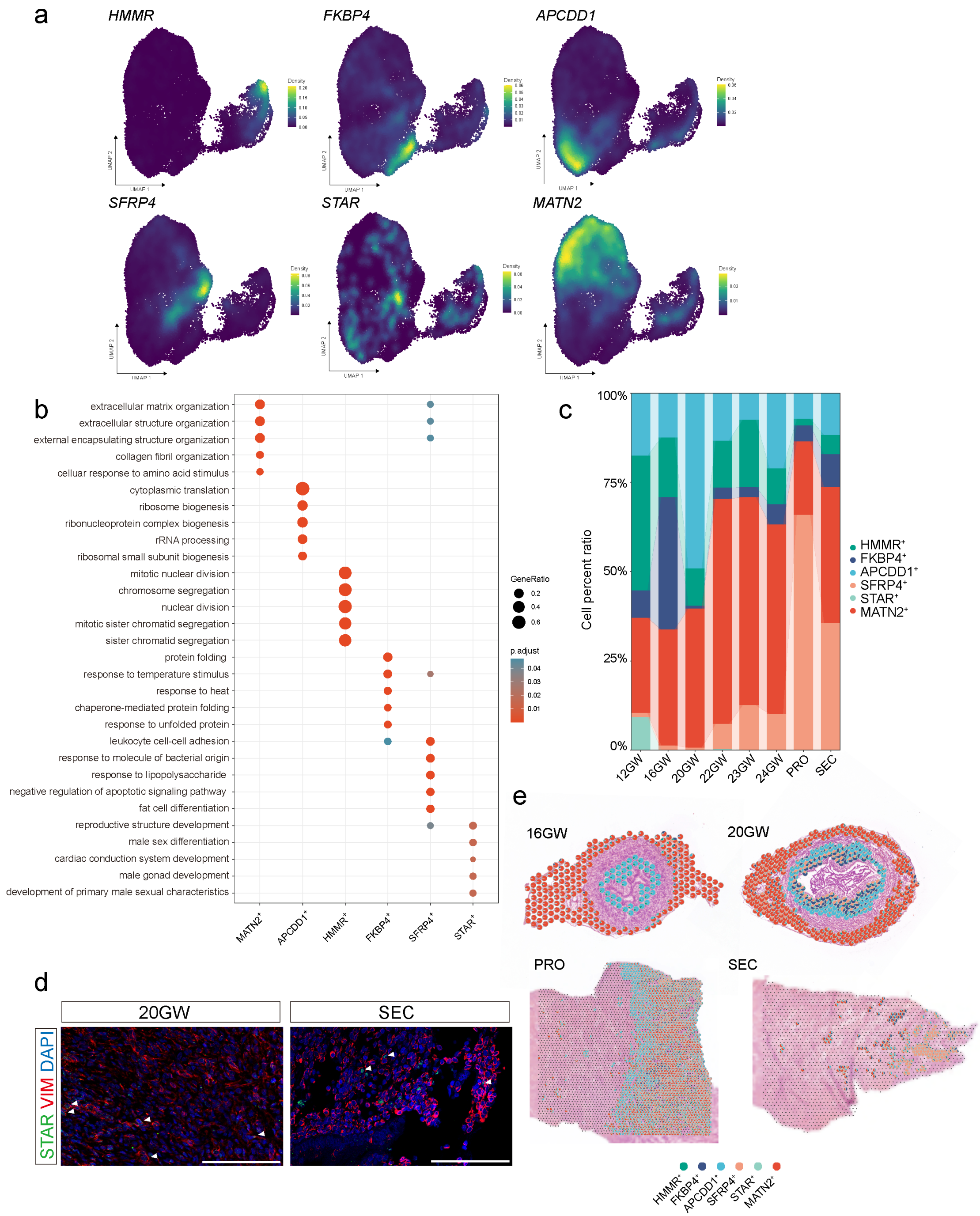

Supplementary Fig S3

Supplementary Fig S3. Characterization of uterine stromal cell subpopulations, related to Fig 2

(a) Plot density depicted classical specific markers for stromal cell subpopulations (*HMMR* for HMMR$^+$ stromal cells; *FKBP* for FKBP$^+$ stromal cells; *APCDD1* for APCDD1$^+$ stromal cells; *SFRP4* for SFRP4$^+$ stromal cells; *STAR* for STAR$^+$ stromal cells; *MATN2* for MATN2$^+$ stromal cells).

(b) Functional enrichment analysis of differentially expressed genes among stromal cell subpopulations.

(c) The proportion of stromal cell subpopulations at different developmental stages.

(d) Immunofluorescence staining of GW20 and adult human uterine sections showing STAR and Vimentin (VIM) co-expression. Arrows indicate STAR$^+$ stromal cells. Scale bars, 100 μm.

(e) Spatial mapping of stromal cell subpopulations in transverse sections of the fetal uterus (GW16, GW20) and full-thickness uterine sections from proliferative (PRO) and secretory (SEC) phases, with colors representing the predicted percentage of each subpopulation per Visium spot based on RCTD deconvolution.

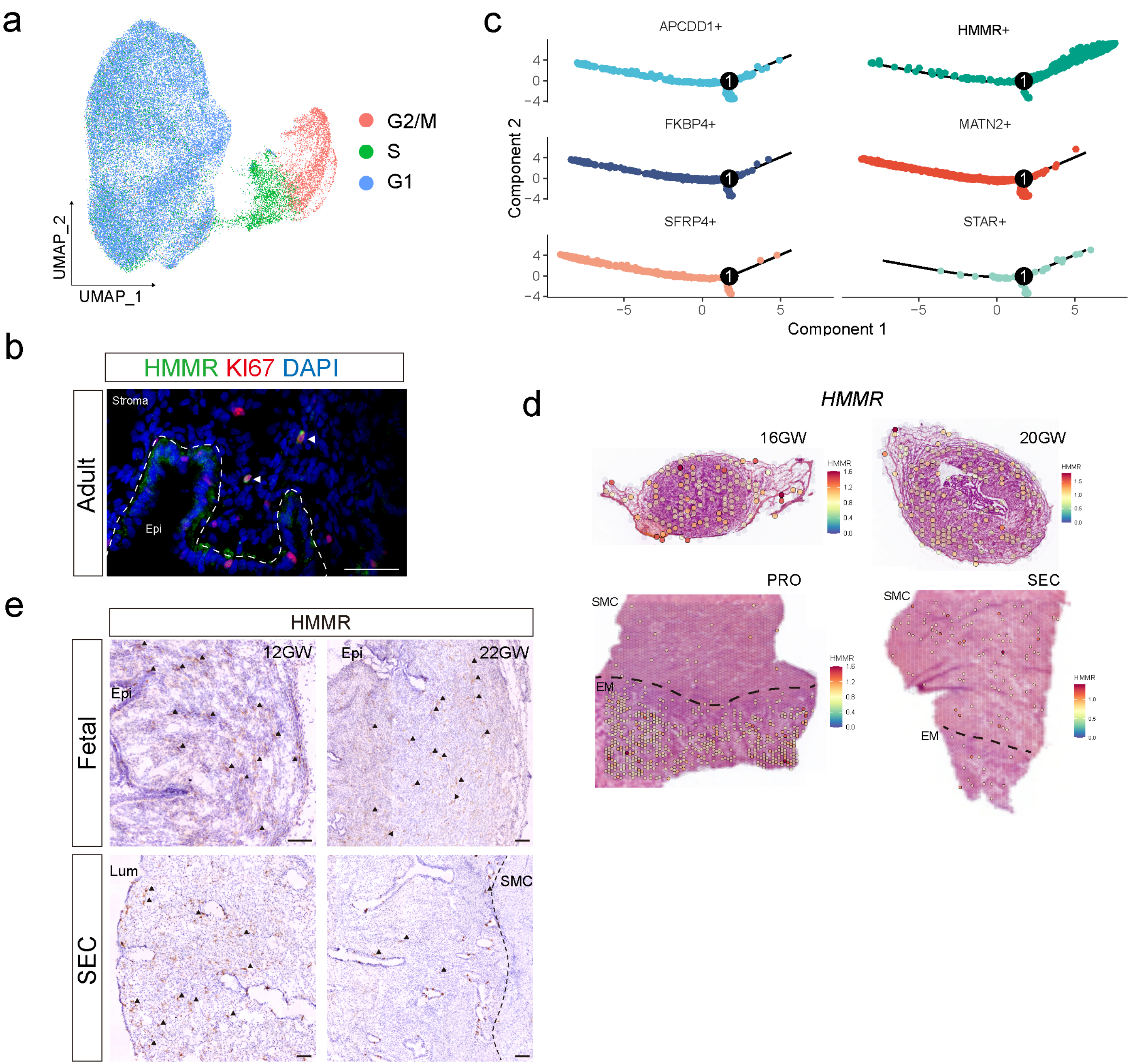

Supplementary Fig S4

Supplementary Fig S4. Characterization of human uterine HMMR$^+$ stromal cell, related to Fig 2

(a) Cell cycle analysis of human uterine stromal cells.

(b) Immunofluorescent staining of adult human uterine sections reveals co-expression of HMMR and KI67. Scale bars, 100 μm. Arrows: positive cells detected by immunofluorescence staining.

(c) Distribution of stromal cell subpopulations along the trajectory constructed by Monocle2.

(d) Spatial mapping of HMMR expression in transverse sections of the fetal uterus (GW16, GW20) and full-thickness uterine sections from proliferative (PRO) and secretory (SEC) phases using Visium spatial transcriptomics. Color intensity represents the predicted *HMMR* expression level per Visium spot.

(e) Immunohistochemical staining of HMMR in human uterine sections at fetal (GW12 and GW22) and secretory (SEC) phases, showing HMMR$^+$ stromal cell localization. Arrows indicate HMMR$^+$ stromal cells. Scale bars, 100 μm.

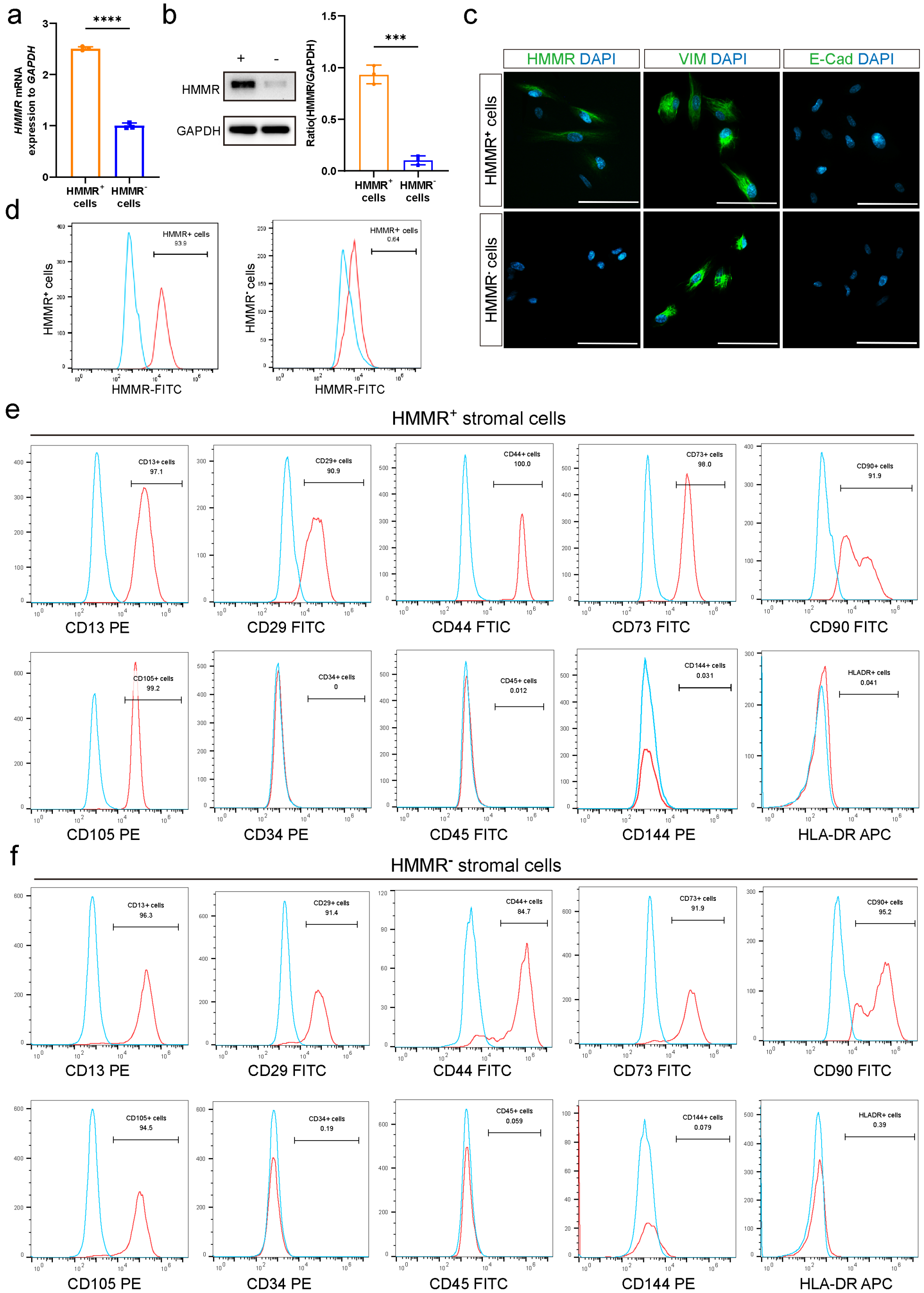

Supplementary Fig S5

Supplementary Fig S5. Identification and immunophenotyping of HMMR$^+$ and HMMR$^-$ stromal cells, related to Fig 3

(a) Gene expression level of *HMMR* normalized to *GAPDH* (n = 3 per group).

(b) Western blot analysis showed that the expression of HMMR was significantly higher in HMMR$^+$ stromal cells than in HMMR$^-$ stromal cells (n = 3 per group).

(c) Immunofluorescence staining of HMMR, Vimentin, and E-cadherin in HMMR$^+$ stromal cells and HMMR$^-$ stromal cells. Scale bars, 100 μm.

(d) Flow cytometric analysis showing the proportion of HMMR$^+$ cells in the populations. Red indicates positive cells; blue indicates negative controls.

(e) Flow cytometric analysis of HMMR$^+$ stromal cells for MSC phenotypes, including CD13, CD29, CD44, CD73, CD90, CD105, CD34, CD45, CD144, and HLA-DR.

(f) Flow cytometric analysis of HMMR$^-$ stromal cells for MSC phenotypes, including CD13, CD29, CD44, CD73, CD90, CD105, CD34, CD45, CD144, and HLA-DR.

Data are expressed as mean ± SD. *P < 0.05, **P < 0.01, ***P < 0.001, ****P < 0.0001, ns, not significant, unpaired t test.

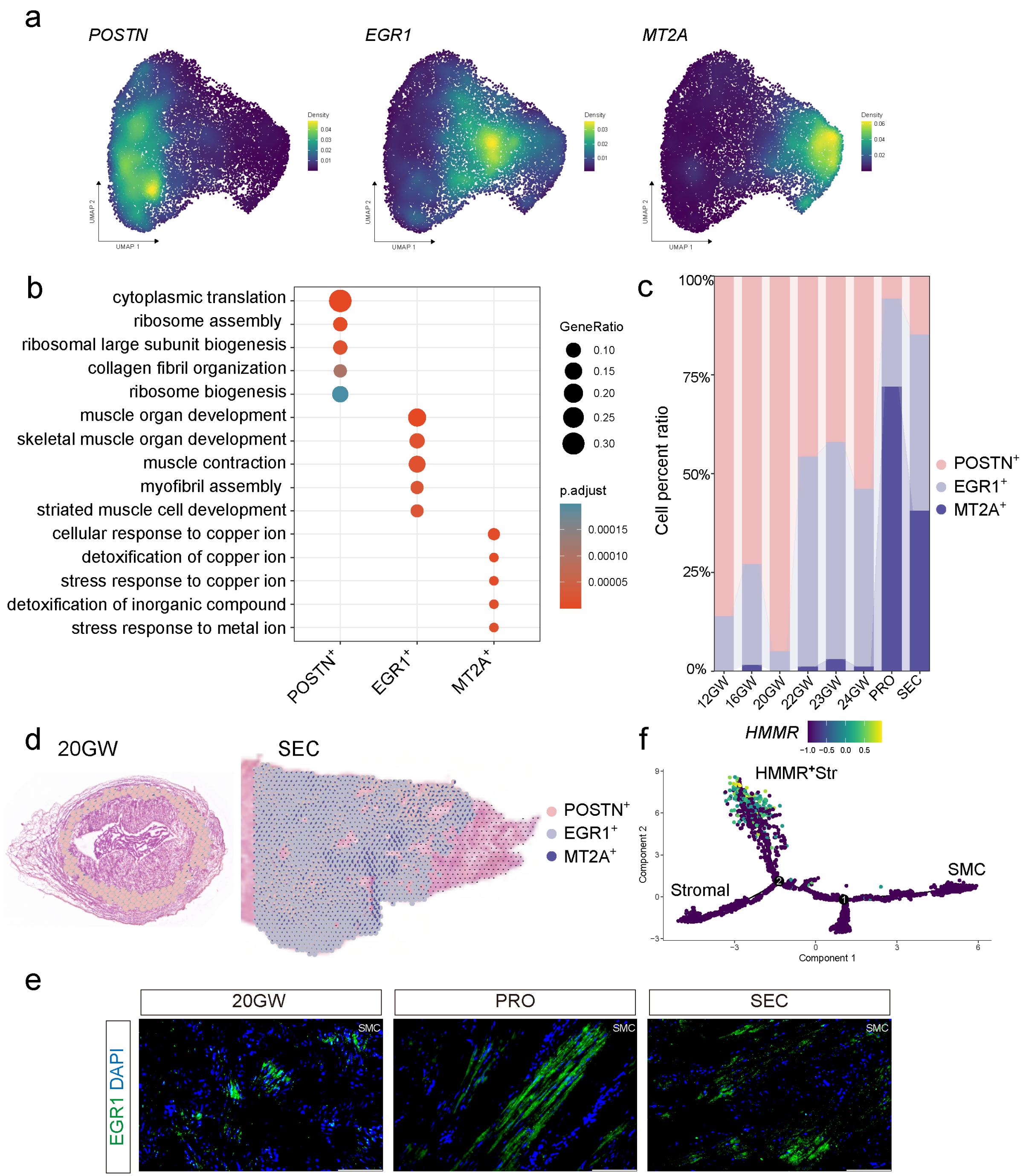

Supplementary Fig S6

Supplementary Fig S6. Characterization of uterine smooth muscle cell subpopulations, related to Fig 5

(a) Plot density depicted classical specific markers for smooth muscle cell subpopulations (*POSTN* for POSTN$^+$ SMCs; *EGR* for EGR1$^+$ SMCs; *MT2A* for MT2A$^+$ SMCs).

(b) Functional enrichment analysis of differentially expressed genes among smooth muscle cell subpopulations.

(c) The proportion of smooth muscle cell subpopulations at different developmental stages.

(d) Spatial mapping of smooth muscle cell (SMC) subpopulations in a transverse section of the fetal uterus (GW20) and a full-thickness uterine section from secretory (SEC) phase, with colors representing the predicted percentage of each SMC subpopulation per Visium spot based on RCTD deconvolution.

(e) Immunofluorescence staining of human uterine sections reveals EGR1 expression at GW20 and in the adult proliferative and secretory phases. Scale bars, 100 μm.

(f) Distribution of HMMR$^+$ stromal cells along the trajectory constructed by Monocle2.

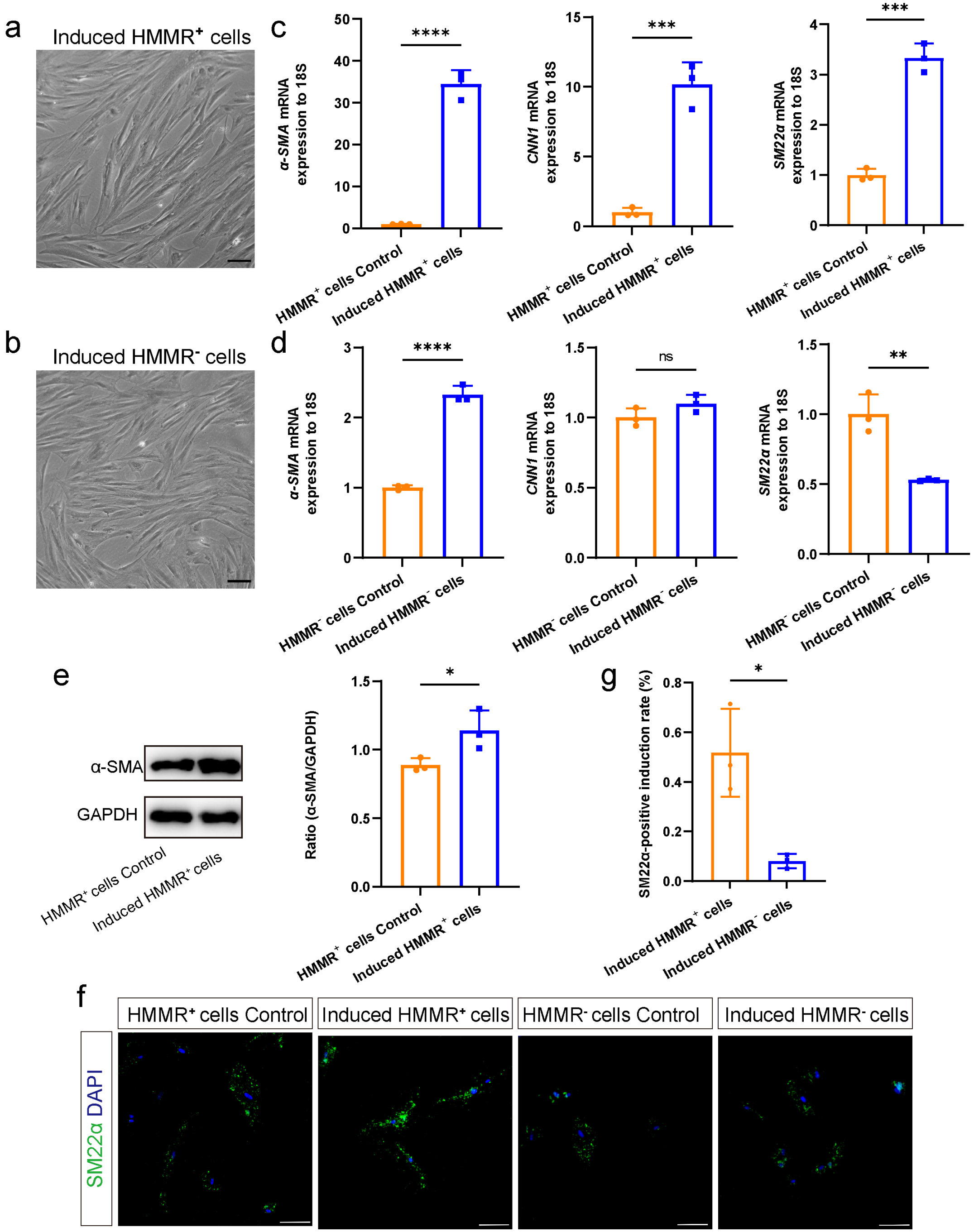

Supplementary Fig S7

Supplementary Fig S7. HMMR$^+$ stromal cells effectively differentiated into smooth muscle cells *in vitro*, related to Fig 5

(a and b) Morphology of HMMR$^+$ and HMMR$^-$ stromal cells cultured *in vitro* following TGF-β induction. Scale bars, 100 μm.

(c) The gene expression levels of SMC markers genes *(αSMA*, *CNN1*, *and SM22α*) in HMMR$^+$ stromal cells before (control) and after induction, normalized to *18S* (n = 3 per group).

(d) The gene expression levels of SMC markers genes (*αSMA*, *CNN1, and SM22α*) in HMMR$^-$ stromal cells before (control) and after induction, normalized to *18S* (n = 3 per group).

(e) Western blot analysis showed that the expression of αSMA was significantly higher in HMMR$^+$ stromal cells before (control) and after induction (n = 3 per group).

(f) Immunofluorescence staining of SM22α was performed in both HMMR$^+$ and HMMR$^-$ stromal cells before (control) and after induction. Scale bars, 100 μm.

(g) Induction rate of SM22α positive cells. HMMR$^+$ cells demonstrated significantly higher induction rate of SM22α positive cells compared to HMMR$^-$ cells.

Data are expressed as mean ± SD. *P < 0.05, **P < 0.01, ***P

< 0.001, ****P < 0.0001, ns, not significant, unpaired t test.

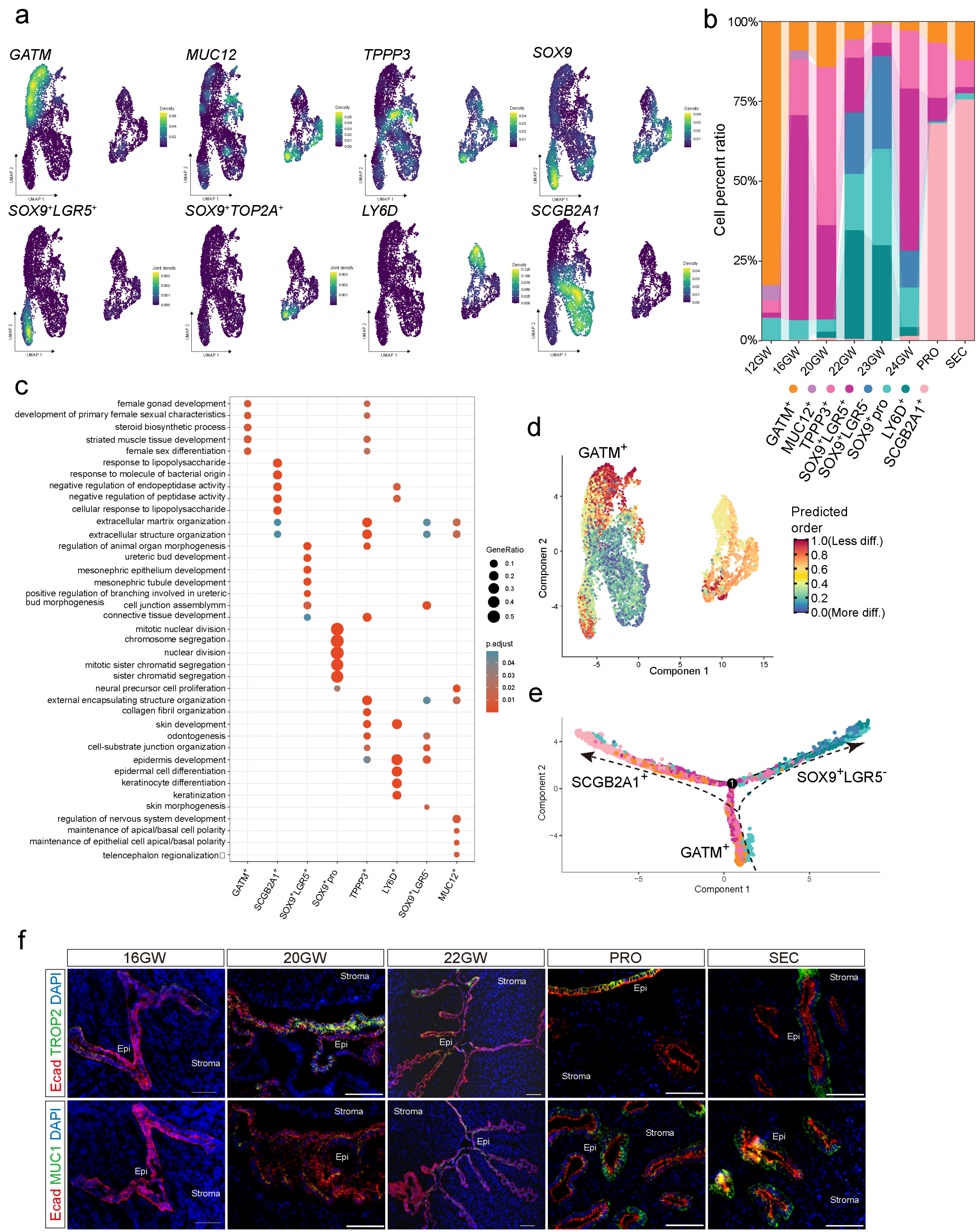

Supplementary Fig S8

Supplementary Fig S8. Characterization and differentiation trajectories of human uterine epithelial cell subpopulations, related to Fig 6

(a) Plot density depicted classical specific markers for epithelial cell subpopulations (*GATM* for GATM$^+$ epithelium; *MUC12* for MUC12$^+$ epithelium; *TPPP3* for TPPP3$^+$ epithelium; *SOX9* and *LGR5* for SOX9$^+$LGR5$^+$ luminal epithelium and SOX9$^+$LGR5$^-$ epithelium; *SOX9* and *TOP2A* for SOX9$^+$ proliferating epithelium; *LY6D* for LY6D$^+$ epithelium; *SCGB2A1* for SCGB2A1$^+$ epithelium).

(b) The proportion of epithelial cell subpopulations at different developmental stages.

(c) Functional enrichment analysis of differentially expressed genes among epithelial cell subpopulations.

(d) UMAP plot showing CytoTRACE differentiation state scores in each epithelial cell subpopulation.

(e) Monocle2 pseudotime trajectory of epithelial cell subpopulations.

(f) Immunofluorescent staining of human uterine sections showing expression of E-cad (epithelial cells) and Trop2 (luminal epithelial cells), and expression of E-cad (epithelial cells) and MUC1 (glandular epithelial cells) at GW16, 20, and

22, as well as in the adult proliferative and secretory phases. Scale bars, 100 μm.

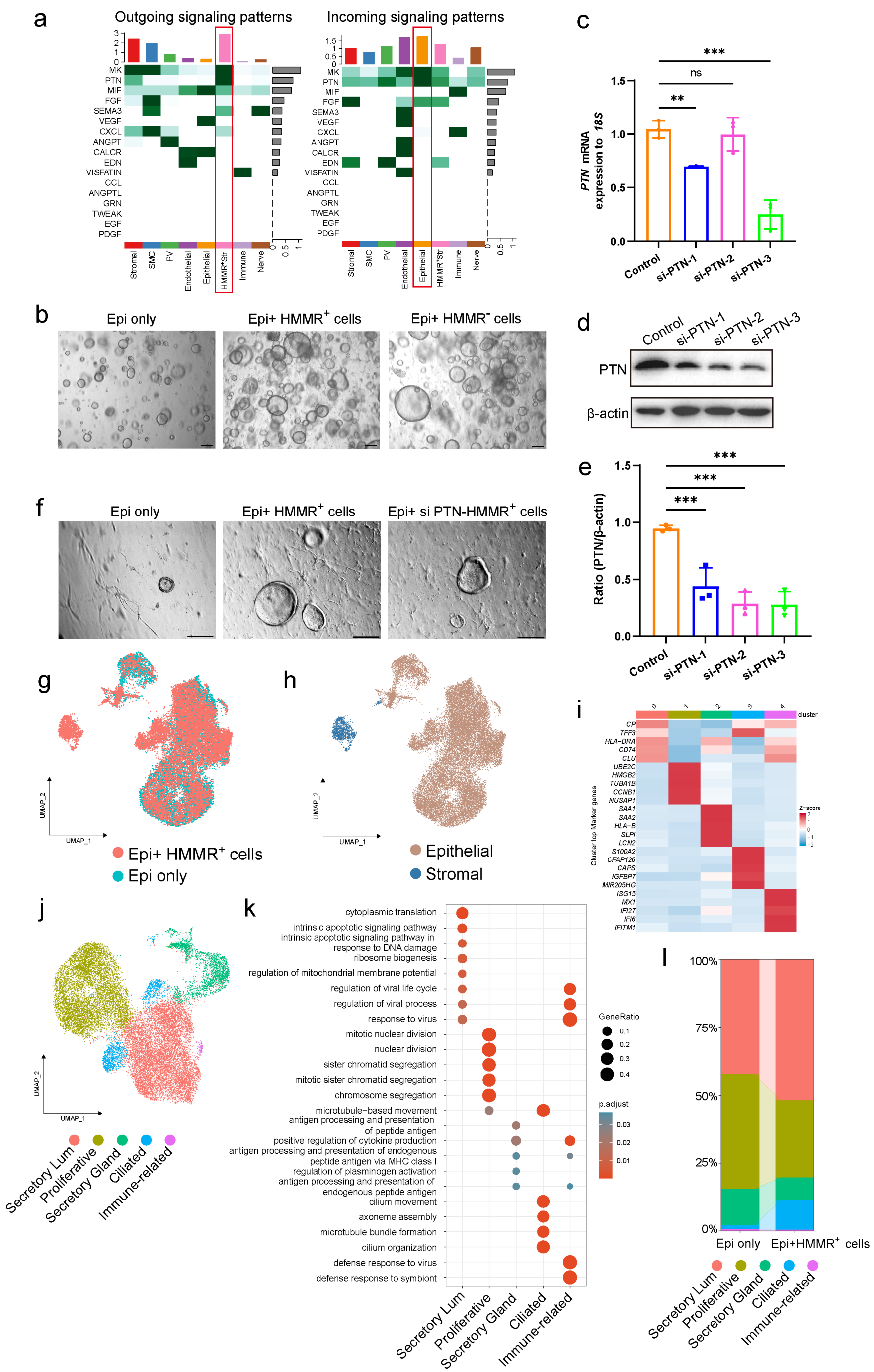

Supplementary Fig S9

Supplementary Fig S9. HMMR$^+$ stromal cells facilitate the growth of endometrial epithelial organoids *in vitro* through PTN signaling, related to Fig 6

(a) During the epithelial developmental stage (fetal), the CellChat analysis diagram of the signals emitted and received from the full-layer uterine cell population.

(b) Organoids culturing of endometrial epithelial cells with HMMR$^+$ or HMMR$^-$ stromal cells co-cultured. Scale bars, 200 μm.

(c) Three different siRNAs were designed targeting distinct regions of human *PTN* mRNA (si-PTN-1, si-PTN-2, si-PTN-3) to knock down *PTN* in HMMR$^+$ stromal cells. The gene expression levels of *PTN* normalized to *18S* (n = 3 per group).

(d and e) Western blot analysis showed the expression of PTN (n = 3 per group).

(f) Organoids culturing of endometrial epithelial cells co-cultured with from control or PTN knockdown (si-PTN) HMMR$^+$ stromal cells. Scale bars, 200 μm.

(g) UMAP plot of all endometrial epithelial organoid cells, colored by culture condition (epithelial cells co-cultured with HMMR$^+$ stromal cells or epithelial cells alone).

(h) UMAP plot of endometrial epithelial organoid cells colored

by cell types.

(i) Heatmap of the top 5 specifically expressed genes for each epithelial subpopulation in cultured endometrial epithelial organoids based on single-cell RNA-seq data.

(j) UMAP plot of the epithelial subpopulations derived from cultured endometrial epithelial organoid single-cell data.

(k) Functional enrichment analysis of differentially expressed genes among epithelial cell subpopulations in endometrial epithelial organoids.

(l) The proportion of epithelial cell subpopulations in endometrial epithelial organoids cultured epithelial cells alone or epithelial cells co-cultured with $HMMR^+$ stromal cells.

Data are expressed as mean ± SD. *$P < 0.05$, **$P < 0.01$, ***$P < 0.001$, ****$P < 0.0001$, ns, not significant, one-way ANOVA with Tukey's multiple comparisons.

Table S1. Antibodies used in this study

Antibody	Source	Concentration
E-cadherin	Cell Signaling Technology 3195S	1:300
E-cadherin	Abcam ab76055	1:300
Vimentin	Abcam ab92547	1:300
CD31	Abcam ab187377	1:300
KI67	Abcam ab279653	1:500
S100B	Abcam ab52642	1:200
TROP-2	Abcam ab214488	1:300
MUC1	Santa Cruz Biotechnology sc-7313	1:100
HMMR	Cell Signaling Technology 87129S	1:300(IF/IHC)
HMMR	Proteintech 15820-1-AP	1:200 (FC)<br>1:1000 (WB)
GAPDH	Bioworld BS42645	1:10000
SFRP4	Abcam ab154167	1:200
STAR	Proteintech 12225-1-AP	1:200
α-SMA	Abcam ab7817	1:500(IF)<br>1:8000(WB)
POSTN	Santa Cruz Biotechnology<br>sc-398631	1:100
MT2A	Abclonal A2018	1:100
EGR1	Abcam ab194357	1:200

anti-rabbit IgG Alexa Fluor 488 conjugate	Abcam ab150077	1:500
anti-rabbit IgG Alexa Fluor 555 conjugate	Abcam ab150078	1:500
anti-mouse IgG Alexa Fluor 488 conjugate	Abcam ab150113	1:500
anti-mouse IgG Alexa Fluor 594 conjugate	Abcam ab150120	1:500
Peroxidase-Conjugated anti-Rabbit IgG	AiFang biological AFIHC001	/
CD45-APC-Cy7	BD Biosciences 557833	1:100
CD56-PE-Cy7	BD Biosciences 557747	1:100
CD144-PerCP-Cy5.5	BD Biosciences 561566	1:100
CD90-PE	BD Biosciences 561970	1:100
CD29-FITC	eBioscience 11-0299-41	1:100
CD73-FITC	eBioscience 11-0739-41	1:100
CD90-FITC	eBioscience 11-0909-41	1:100
CD13-PE	eBioscience 12-0138-42	1:100
CD44-FITC	BD Pharmingen 560977	1:100
CD34-PE	Multisciences F1103402	1:100
CD45-FITC	eBioscience 11-0453-81	1:100
HLA-DR-APC	Multisciences F110HD03	1:100
CD105-PE	eBioscience 12-1057-42	1:100
CD144-PE	BD Pharmingen 561714	1:100

Table S2. Sequences of primers used for qRT-PCR analysis

	Forward Primer	Reverse Primer
HMMR	CTGAAAGGGAAGGAGGCTGA	TCTCACTGGCTGTTAACGCT
KI67	GTCCTCTCTGTGTTCCCCAA	CTGGGGAAACAAACGTGGAG
PCNA	AGGCACTCAAGGACCTCATC	GCCAAGGTATCCGCGTTATC
SFRP4	CCTATCAAGCCGTGCAAGTC	TCCACTTAACATCCTCCGGG
GAPDH	CCAAGGAGTAAGACCCCTGG	TGGTTGAGCACAGGGTACTT
α-SMA	ACCCAGCACCATGAAGATCA	AGAGACAGAGAGGAGCAGGA
Calponin-1	ACGACCCCAAGTACTGTCTG	GGTTGAGTTCTACAGGGGCT
SM22-α	TATGGCAGGCACCTTCTCAA	AGGATTGCTGCCAGAGAAGT
PTN	GGAGCTGAGTGCAAGCAAAC	CTCGCTTCAGACTTCCAGTTC
18S	AGAAACGGCTACCACATCCA	CCCTCCAATGGATCCTCGTT
